# Fetal Loss in Pregnant Rabbits Infected with Genotype 3 Hepatitis E Virus Is Associated with Altered Inflammatory Responses, Enhanced Virus Replication, and Extrahepatic Virus Dissemination with Positive Correlations with Increased Estradiol Level

**DOI:** 10.1128/mbio.00418-23

**Published:** 2023-03-20

**Authors:** Hassan M. Mahsoub, C. Lynn Heffron, Anna M. Hassebroek, Harini Sooryanarain, Bo Wang, Tanya LeRoith, Guillermo Raimundi Rodríguez, Debin Tian, Xiang-Jin Meng

**Affiliations:** a Department of Biomedical Sciences and Pathobiology, Virginia-Maryland College of Veterinary Medicine, Virginia Polytechnic Institute and State University, Blacksburg, Virginia, USA; b Center for Emerging, Zoonotic and Arthropod-borne Pathogens, Fralin Life Sciences Institute, Virginia Polytechnic Institute and State University, Blacksburg, Virginia, USA; Indiana University Bloomington

**Keywords:** hepatitis E virus (HEV), genotype 3 HEV, rabbit HEV, estradiol, pregnancy, fetal loss, inflammatory cytokines

## Abstract

Hepatitis E virus (HEV) causes adverse clinical outcomes in pregnant women, but the underlying mechanisms remain poorly understood. To delineate the mechanisms of pregnancy-associated adverse effects during HEV infection, we utilized a genotype 3 HEV from rabbit (HEV-3ra) and its cognate host (rabbits) to systematically investigate the clinical consequences, viral replication dynamics, and host immune and hormonal responses of HEV infection during pregnancy. We found a significant fetal loss of 23% in HEV-infected pregnant rabbits, indicating an early-stage miscarriage. HEV infection in pregnant rabbits was characterized by higher viral loads in feces, intestinal contents, liver, and spleen tissues, as well as a longer and earlier onset of viremia than in infected nonpregnant rabbits. HEV infection altered the pattern of cytokine gene expressions in the liver of pregnant rabbits and caused a transient increase of serum interferon gamma (IFN-γ) shortly after a notable increase in viral replication, which may contribute to early fetal loss. Histological lesions in the spleen were more pronounced in infected pregnant rabbits, although moderate liver lesions were seen in both infected pregnant and nonpregnant rabbits. Total bilirubin was elevated in infected pregnant rabbits. The serum levels of estradiol (E2) in HEV-infected pregnant rabbits were significantly higher than those in mock-infected pregnant rabbits at 14 days postinoculation (dpi) and correlated positively with higher viral loads in feces, liver, and spleen tissues at 28 dpi, suggesting that it may play a role in extrahepatic virus dissemination. The results have important implications for understanding the severe diseases associated with HEV infection during pregnancy.

## INTRODUCTION

Hepatitis E virus (HEV), the causative agent of hepatitis E, is a leading cause of acute viral hepatitis worldwide and has emerged as a global public health concern in recent years (1), with an estimate of 20.1 million infections, 3.4 million symptomatic cases, and >70,000 deaths occurring annually (2). HEV typically causes an acute, self-limiting infection with a mortality rate of 0.5% to 3% in the general population (3), and the healthy host immune system clears the virus infection within a few weeks of its onset. However, in immunosuppressed individuals, such as recipients of solid organ transplants and HIV-infected patients, HEV can cause chronic infections that may progress to fatal liver diseases as well as extrahepatic manifestations of diseases (4–9). Chronic hepatitis E was also reported in immunocompetent patients (10–12).

In pregnant women, adverse effects of HEV-1 infection include vertical virus transmission of >30%, preterm delivery, miscarriage, stillbirth (an estimate of 3,000 cases annually), and fulminant hepatic failure (FHF), with a mortality rate of up to 30% (2, 13–17). Recent reports from several countries, including Nigeria (18), Senegal (19), Thailand (20), Egypt (21), and Argentina (22), showed that HEV infections in pregnant women continue to be a more significant clinical problem than in nonpregnant women and immunocompetent individuals. Among the hepatotropic viruses, the high mortality in pregnant women is unique to HEV, and therefore, there is a critical need for an appropriate animal model to delineate the underlying mechanisms of HEV-induced adverse outcomes during pregnancy. Unfortunately, attempts to develop an animal model using pregnant rhesus macaques (23) and pregnant sows (24) that could reproduce the severe diseases observed in infected pregnant women were unsuccessful.

HEV is a nonenveloped virus, with a single-stranded, positive-sense RNA genome of ~7.2 kb and three partially overlapping open reading frames (ORFs) (25–27), although the virus in circulating blood exists as exosome-like quasi-enveloped infectious particles (28–30). The ORF1 encodes nonstructural proteins, ORF2 encodes capsid proteins (29), and ORF3 encodes multifunctional iron channel proteins (31, 32). HEV is classified in the family of *Hepeviridae*, which contains two subfamilies, *Orthohepevirinae* and *Parahepevirinae*. The *Orthohepevirinae* subfamily consists of four distinct genera containing members that infect avian species (*Avihepevirus*); bats (*Chirohepevirus*); humans and other mammals (*Paslahepevirus*); and rodents, shrews, and carnivores (*Rocahepevirus*). The *Parahepevirinae* subfamily comprises only one genus (*Piscihepevirus*) of viruses that infect fish (33, 34). The primary species, *balayani*, within the genus *Paslahepevirus* contains eight distinct genotypes, of which HEV-1 and HEV-2 exclusively infect humans, causing major outbreaks in developing countries; HEV-3 and HEV-4 are zoonotic and infect humans and several other animal species, causing sporadic and cluster cases in many industrialized countries (35, 36).

Variant strains of HEV were isolated from rabbits in China, the United States, and several other countries (37–40). Sequence and phylogenetic analyses revealed that all rabbit HEV variants belong to HEV-3 and therefore were designated HEV-3ra (33, 41). Under experimental conditions, HEV-3ra infects rabbits (42, 43), pigs, and cynomolgus macaques and also replicates in human cell lines (42, 44–47). Incidences of HEV-3ra infections in humans have also recently been reported in several European countries (48–50), and rabbits are considered an animal reservoir for zoonotic HEV infection in humans (36, 51). Pregnant rabbits experimentally infected with HEV reportedly had adverse outcomes (fetal mortality, miscarriages, stillbirths, and fulminant hepatitis) similar to those reported in pregnant women (52–54), but the underlying mechanisms leading to these reported adverse outcomes remain largely unknown.

In this study, we utilized an HEV-3ra infection of pregnant rabbit model to systematically investigate the adverse clinical outcomes associated with HEV infection during pregnancy and delineate the potential hormonal and immunological underlying mechanisms that may contribute to the severe diseases during pregnancy.

## RESULTS

### HEV-3ra infection causes fetal loss in pregnant rabbits.

**FIG 1:**
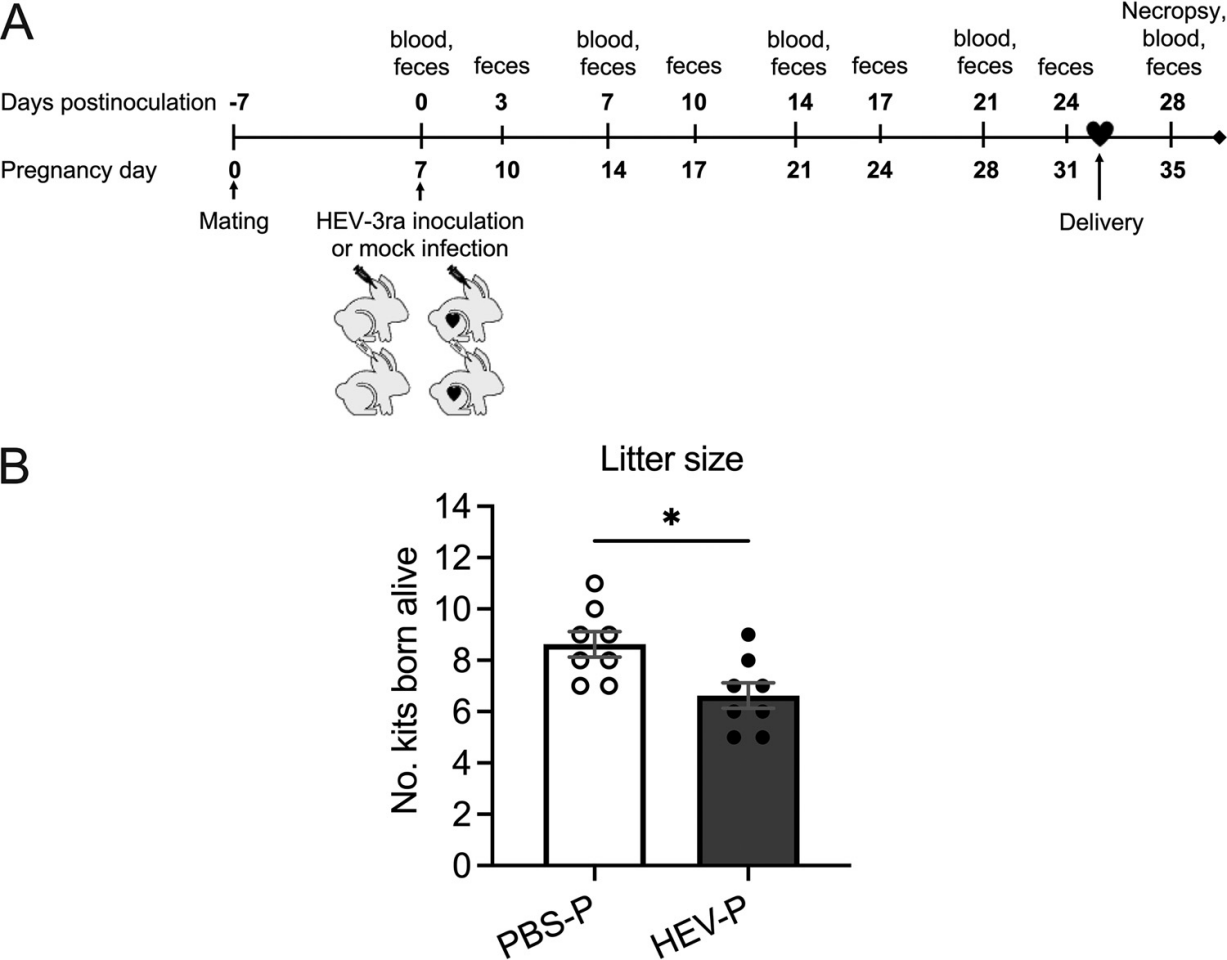
Fetal loss in HEV-3ra-infected pregnant rabbits. (A) Schematic of the experimental design for HEV-3ra infection in pregnant and nonpregnant rabbits. Specific-pathogen-free (SPF) pregnant (at 7 days of gestation) and nonpregnant female New Zealand White rabbits were intravenously inoculated via the ear vein with HEV-3ra (two groups; *n* = 8) and PBS as mock infection (two groups, *n* = 8). Fecal samples were collected twice weekly, and serum samples were collected weekly from each rabbit. All rabbits were necropsied at 28 days postinoculation (dpi). (B) Litter size in pregnant rabbits experimentally infected with HEV-3ra or mock-infected with PBS buffer. HEV-infected pregnant rabbits had a significant fetal loss of 23% compared to the mock-infected pregnant rabbits. Statistical significance was determined by two-tailed unpaired Student's *t* test (*, *P < *0.05). Bars indicate mean ± SEM. HEV-P, HEV-infected pregnant rabbits (*n* = 8); PBS-P, uninfected pregnant rabbits (*n* = 8).

HEV infection in humans during pregnancy is associated with FHF and higher mortality (13, 55). By utilizing HEV-3ra and a pregnant rabbit model (52, 53), we systematically investigated the adverse clinical outcomes and the potential hormonal and immunological underlying mechanisms associated with HEV infection during pregnancy. Four groups of age-matched pregnant and nonpregnant rabbits (*n* = 8) were either infected with HEV-3ra at 7 days of gestation or mock-inoculated with phosphate-buffered saline (PBS) (Table 1; Fig. 1A). We found that HEV-infected pregnant (HEV-P) rabbits had a significantly (*P = *0.0131) reduced number of kits born alive (mean, 6.63 kits; median, 6.5 kits/litter) compared to uninfected pregnant rabbits (PBS-P; mean, 8.63; median, 8.5 kits/litter), indicating a fetal loss of 23.19% due to HEV-3ra infection (Fig. 1B). Rabbit, like mouse, is a multiparous animal, and intrauterine fetal death or absorption can be considered a miscarriage (56). None of the pregnant rabbits in both groups had noticeable birth complications or late-stage abortion/miscarriage, and thus, the fetal loss likely occurred during the early stages of pregnancy before the complete formation of fetuses in the uterus. In the HEV-P group, 2 rabbits (rabbits 31 and 34) had 1 stillbirth each, along with 6 kits born alive; while in the control group, 1 rabbit (rabbit 38) had 1 stillbirth, along with 10 kits born alive (see Table S1 in the supplemental material).

**TABLE 1:** Experimental infection of pregnant and nonpregnant New Zealand White female rabbits with HEV-3ra

Group	Pregnancy status	Inoculum^*a*^	No. of rabbits for sampling and necropsy at 28 dpi
PBS-NP	Nonpregnant	PBS	8
PBS-P	Pregnant	PBS	8
HEV-NP	Nonpregnant	HEV-3ra	8
HEV-P	Pregnant	HEV-3ra	8

a Rabbits were intravenously inoculated with HEV-3ra or PBS (mock) at 7 days of gestation (in pregnant rabbit groups).

10.1128/mbio.00418-23.3 TABLE S1 Numbers of kits born alive and the numbers of stillbirths per litter in mock (PBS)-inoculated and HEV-3ra-inoculated pregnant rabbit groups. Download Table S1, DOCX file, 0.01 MB. Copyright © 2023 Mahsoub et al. 2023 Mahsoub et al. https://creativecommons.org/licenses/by/4.0/ This content is distributed under the terms of the Creative Commons Attribution 4.0 International license.

### Higher fecal viral RNA loads in HEV-3ra-infected pregnant rabbits than in nonpregnant rabbits.

**FIG 2:**
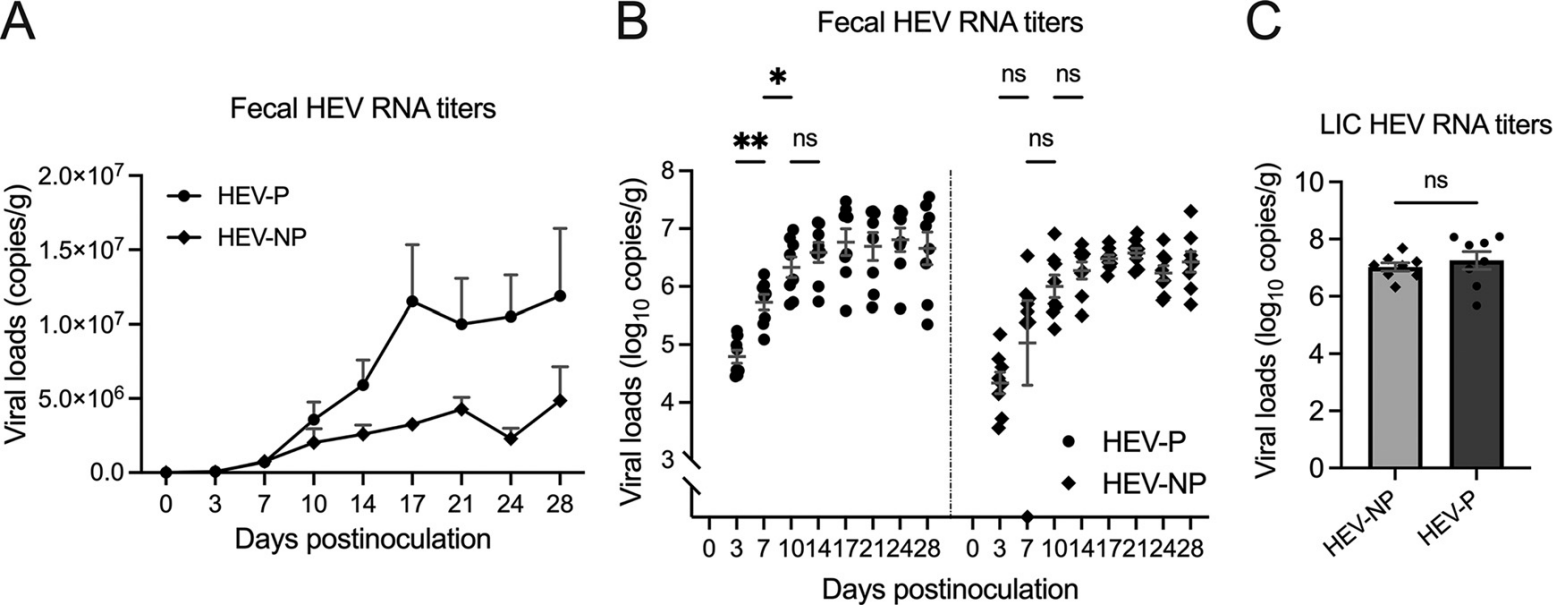
Fecal virus shedding in pregnant and nonpregnant female rabbits experimentally infected with HEV-3ra. (A) Kinetics of viral RNA loads in fecal samples during the course of HEV-3ra infection in pregnant and nonpregnant rabbits. HEV RNA loads were quantified by RT-qPCR in twice-weekly fecal samples at the indicated days postinoculation (dpi). Numerical values of HEV RNA copies/g are indicated. (B) Fecal viral RNA titers in panel A were converted to log_10_ values, and data are presented as a scatterplot showing viral RNA loads of each individual rabbit with statistical analyses of viral RNA titers between the different time points postinoculation within the infected pregnant and infected nonpregnant groups. In the HEV-infected pregnant rabbit group, fecal viral RNA titers significantly increased during the 3- to 7-dpi and 7- to 10-dpi time intervals (*P = *0.0050 and 0.0498, respectively). (C) Viral RNA loads in large intestinal content (LIC) collected at necropsy at 28 dpi from infected pregnant and nonpregnant rabbits. Differences in fecal viral RNA titers among the different time points within each group were statistically analyzed by two-way ANOVA, and asterisks indicate statistical significance. Bars indicate mean ± SEM. HEV-P, HEV-infected pregnant rabbits (*n* = 8); HEV-NP, HEV-infected nonpregnant rabbits (*n* = 8); ns, not significant; *, *P < *0.05; **, *P < *0.01.

Viral RNA loads in the twice-weekly fecal samples were numerically higher in HEV-infected pregnant rabbits (HEV-P) than in HEV-infected nonpregnant rabbits (HEV-NP) (Fig. 2A; Table S2), although the differences were not statistically significant at the different time points. In both HEV-P and HEV-NP groups, fecal virus shedding began as early as at 3 dpi (7.84 × 10^4^ genome copies/g feces for HEV-P and 3.96 × 10^4^ genome copies/g feces for HEV-NP) and remained positive until necropsy at 28 dpi, except for one HEV-NP rabbit, which tested negative at 7 dpi but was positive thereafter (Fig. 2A). At 3 dpi, the average viral RNA load of the HEV-P group was 0.98-fold higher than that of the HEV-NP group. The difference between the two groups peaked at 24 dpi when the viral load in HEV-P rabbits was 3.57-fold higher. The fecal viral RNA loads in HEV-P rabbits gradually increased from 3 dpi to 24 dpi before dropping slightly at 28 dpi. In HEV-NP rabbits, viral RNA loads increased from 3 dpi to 21 dpi and then dropped between 24 and 28 dpi (Fig. 2A and B). The viral RNA loads in large intestinal contents (LIC) collected at necropsy at 28 dpi were also numerically higher (2.34-fold higher) in HEV-P rabbits than in HEV-NP rabbits (Fig. 2C; Table S2), but the difference was not statistically different.

10.1128/mbio.00418-23.4 TABLE S2 Viral RNA load data (log_10_ copies of HEV RNA/gram) in fecal and large intestinal content (LIC) samples collected from HEV-3ra-infected pregnant (HEV-P) and nonpregnant (HEV-NP) rabbits. Download Table S2, DOCX file, 0.02 MB. Copyright © 2023 Mahsoub et al. 2023 Mahsoub et al. https://creativecommons.org/licenses/by/4.0/ This content is distributed under the terms of the Creative Commons Attribution 4.0 International license.

We further analyzed and compared the fecal viral shedding data from 3 dpi to 28 dpi within each of the HEV-P and HEV-NP groups. The two-way analysis of variance (ANOVA) of the fecal viral RNA loads showed that the “infection × pregnancy” interaction was not significant; however, “time postinoculation” had a significant effect (*P < *0.0001). This indicates that fecal viral loads differed significantly among certain time points within the treatment group. Tukey’s *post hoc* analysis showed that, in the HEV-P group, fecal viral loads significantly increased during the 3- to 7-dpi and 7- to 10-dpi time intervals (*P = *0.0050 and 0.0498, respectively) (Fig. 2B). On the contrary, in the HEV-NP group, the increase in fecal viral loads during these same intervals was not statistically significant (*P = *0.7904 and 0.7230, respectively). The increase in viral loads during subsequent intervals was not significant in both groups. Overall, the data show that HEV infection caused higher levels of virus replication in pregnant rabbits during the early stages of gestation than in nonpregnant rabbits. It is plausible that the increase in viral replication at the early stages of HEV infection in pregnant rabbits, along with other unfavorable virus-induced systemic and localized inflammatory responses, may contribute to the observed early fetal loss.

### Earlier onset and longer duration of viremia in HEV-3ra-infected pregnant rabbits than in nonpregnant rabbits.

**FIG 3:**
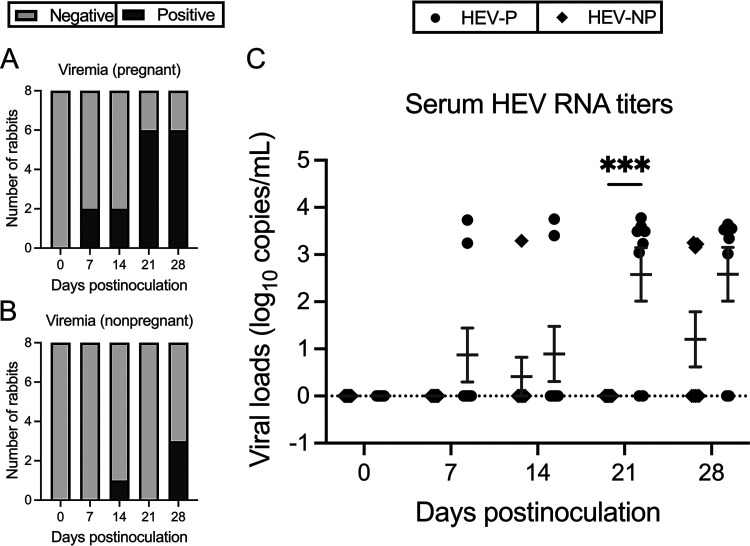
Comparison of serum viral RNA titers, incidence, and duration of viremia in pregnant and nonpregnant rabbits experimentally infected with HEV-3ra. (A and B) Numbers (incidence) of HEV viremia-positive and HEV viremia-negative rabbits at different days postinoculation (dpi) in HEV-3ra-infected pregnant and infected nonpregnant rabbits, respectively. (C) Quantification of HEV RNA loads in weekly serum samples collected at 0, 7, 14, 21, and 28 dpi (necropsy) from pregnant and nonpregnant rabbits experimentally infected with HEV-3ra. Total RNA was extracted from the weekly serum samples, and viral RNA copies were quantified by RT-qPCR. Data are presented as a scatterplot, with each symbol indicating the viral RNA titer of an individual rabbit. Statistical significance was determined by two-way ANOVA (***, *P < *0.001). Bars indicate mean ± SEM. HEV-P, HEV-infected pregnant rabbits (*n* = 8); HEV-NP, HEV-infected nonpregnant rabbits (*n* = 8).

The amounts of HEV RNAs in sera from each rabbit were quantified in weekly serum samples by reverse transcriptase quantitative PCR (RT-qPCR). In HEV-P rabbits, viremia was detected as early as at 7 dpi in 2/8 rabbits, which remained positive at necropsy at 28 dpi. At 21 dpi, 4 more rabbits became viremic, bringing the total to 6/8 viremia-positive rabbits (75%). By 28 dpi, one rabbit became negative and one new rabbit tested positive, maintaining 6/8 pregnant rabbits with viremia (Fig. 3A). In HEV-NP rabbits, viral RNA was only detected in the serum of 3/8 (37.5%) rabbits; one rabbit had intermittent viremia at 14 and 28 dpi, and two rabbits tested positive at necropsy at 28 dpi (Fig. 3B). Because of the low numbers of rabbits that tested positive in both HEV-NP and HEV-P groups at 7 and 14 dpi and in the HEV-NP group at 28 dpi (which led to high individual variations within that group), there is no significant difference in viral loads in sera between the two groups at these time points (Fig. 3C). At 14 dpi, the average viral loads in sera of the two positive HEV-P rabbits was 1.08-fold higher than that of the positive HEV-NP rabbit. At 28 dpi, the average serum viral load for the six positive HEV-P rabbits was 0.89-fold higher than that of the three positive HEV-NP rabbits. At 21 dpi, the mean viral load in sera of HEV-P rabbits was significantly higher (*P < *0.001) than that of HEV-NP rabbits, which all tested negative (Fig. 3C). The viral load data from individual serum samples at the different time points postinoculation is included in Table S3.

10.1128/mbio.00418-23.5 TABLE S3 Viral load data (log_10_ copies of HEV RNA/mL or gram) in serum, bile, and tissue samples collected from HEV-3ra-infected pregnant (HEV-P) and nonpregnant (HEV-NP) rabbits. Download Table S3, DOCX file, 0.02 MB. Copyright © 2023 Mahsoub et al. 2023 Mahsoub et al. https://creativecommons.org/licenses/by/4.0/ This content is distributed under the terms of the Creative Commons Attribution 4.0 International license.

### Higher hepatic and extrahepatic viral loads and higher extrahepatic virus dissemination in HEV-3ra-infected pregnant rabbits than in nonpregnant rabbits.

**FIG 4:**
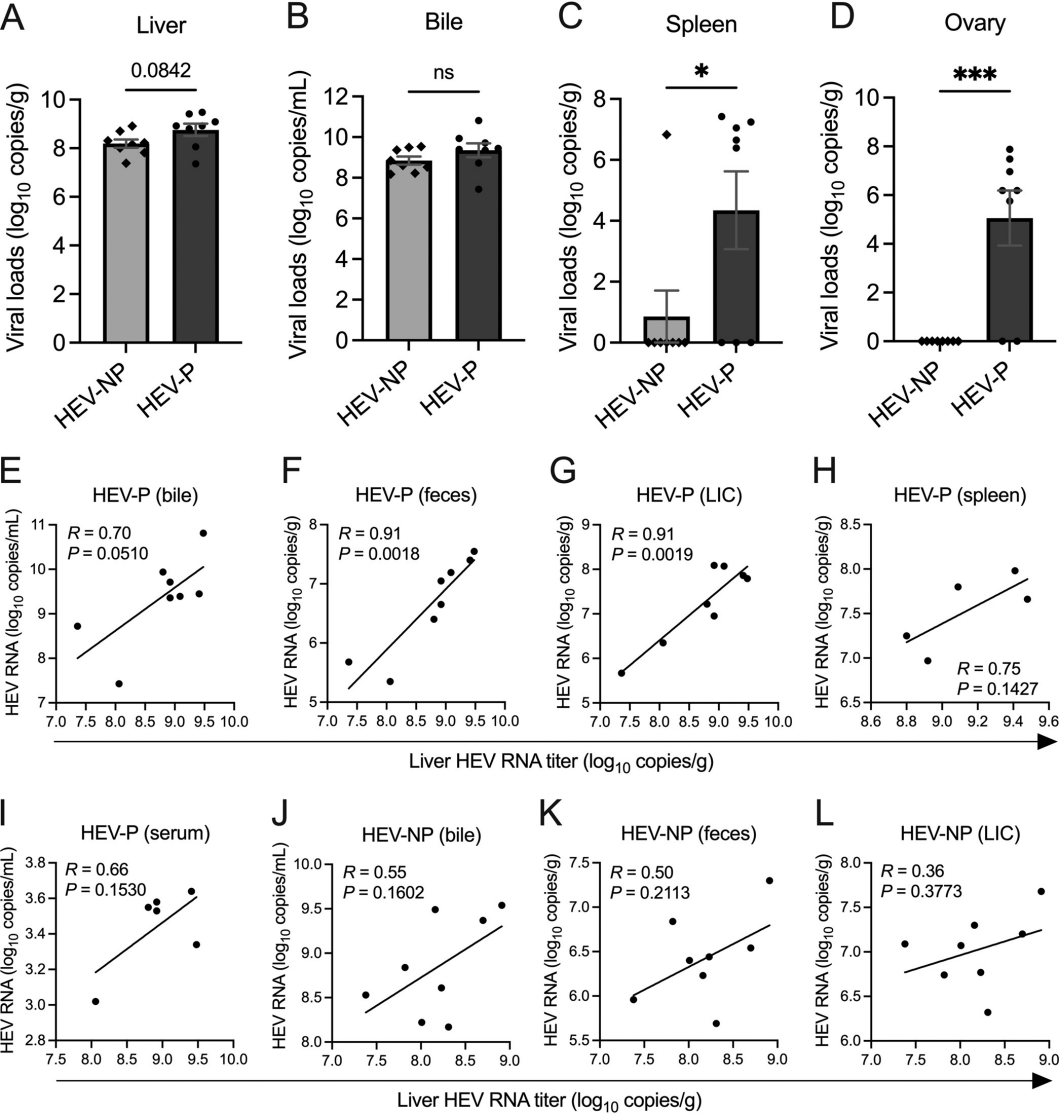
Viral RNA loads in different tissues of HEV-3ra-infected pregnant and nonpregnant rabbits. (A to D) HEV RNA loads in liver, bile, spleen, and ovary tissues collected at necropsy at 28 dpi from infected pregnant and nonpregnant rabbits. Total RNA was extracted from various tissue samples, and viral RNA copies were quantified by RT-qPCR. Each symbol represents HEV RNA copy number per gram tissue from an individual rabbit. Asterisks indicate statistical significance between the two tested groups as determined by two-tailed unpaired Student's *t* test (ns, not significant; *, *P < *0.05; ***, *P < *0.001). Bars indicate mean ± SEM. (E to L) Statistical correlation between viral RNA loads in liver and viral RNA loads in other tissue and nontissue samples at 28 dpi were determined in HEV-infected pregnant and nonpregnant rabbits. The Pearson’s correlation test *P* value and *R* coefficient are indicated in each graph. HEV-P, HEV-infected pregnant rabbits; HEV-NP, HEV-infected nonpregnant rabbits.

During acute HEV infection, the virus replicates in target cells, leading to viremia and subsequent spreading into extrahepatic tissues (57, 58). Since HEV-P rabbits had higher incidences of viremia, we quantified viral loads in liver, bile, and extrahepatic tissues (spleen, ovary, and kidney) collected at necropsy at 28 dpi, as well as in placenta tissues collected at delivery (Table 2; Table S3). We found that the average viral loads in liver and bile were 3.59-fold and 7.08-fold higher in the HEV-P group than those in the HEV-NP group, respectively, indicating a higher virus replication level in pregnant rabbits than in nonpregnant ones. However, these differences between the two groups were not statistically significant (*P = *0.0842 and 0.2254, respectively) due to higher individual rabbit variations in the HEV-P group (Fig. 4A and B). Notably, there was one bile sample and one liver sample in the HEV-P group with very low viral RNA loads (i.e., 2.71 × 10^7^ and 2.31 × 10^7^ copies/mL or g, respectively), which prevented the differences from being statistically significant. Extrahepatically, HEV RNA was detected in spleen and ovary tissues, with substantially more positive samples in the HEV-P rabbits (5/8 and 6/8, respectively) than in the HEV-NP rabbits (1/8 and 0/8, respectively) (Table 2). Viral loads in the spleen and ovary of pregnant rabbits were significantly higher than those in nonpregnant rabbits (*P < *0.05 and *P* < 0.001) (Fig. 4C and D, respectively). Placenta samples in 3/8 of infected pregnant rabbits tested positive for HEV RNA, with an average viral load of 8.25 × 10^3^ copies/g. Kidney samples from all rabbits tested negative for HEV RNA (Table 2).

**TABLE 2:** Detection of HEV RNA in various tissue and bile samples collected at necropsy at 28 dpi from pregnant and nonpregnant rabbits experimentally inoculated with HEV-3ra

Group	Pregnancy status	Inoculum	No. of HEV RNA-positive samples/total no. tested in:					
			Liver	Spleen	Ovary	Placenta	Kidney	Bile
PBS-NP	Nonpregnant	PBS	0/8	0/8	0/8	NA	0/8	0/8
PBS-P	Pregnant	PBS	0/8	0/8	0/8	0/8	0/8	0/8
HEV-NP	Nonpregnant	HEV-3ra	8/8	1/8	0/8	NA	0/8	8/8
HEV-P	Pregnant	HEV-3ra	8/8	5/8	6/8	3/8	0/8	8/8

Pearson’s correlations were estimated between viral loads in liver and those in bile, fecal, and other tissue samples (spleen and ovary) to determine whether the magnitude of viral replication in liver affects extrahepatic viral dissemination. In HEV-P rabbits, viral loads in liver had a near-significant positive correlation with those in bile (*r* = 0.70, *P = *0.0510) (Fig. 4E) and significant positive correlations with those in feces at 28 dpi (*r* = 0.91, *P = *0.0018) and LIC (*r* = 0.91, *P = *0.0019) (Fig. 4F and G, respectively). Positive, but not statistically significant, correlations were also found between viral loads in the liver and those in spleen (*r* = 0.75, *P* = 0.1427) and serum (*r* = 0.66, *P* = 0.1530) (Fig. 4H and I, respectively). In HEV-NP rabbits, only weak positive correlations were found between viral loads in the liver and those in bile, feces, and LIC (*r* = 0.55, 0.50, and 0.36) (Fig. 4J to L, respectively). These results indicate that HEV-infected pregnant women are more likely to suffer the consequences of extrahepatic virus dissemination than nonpregnant ones, which may eventually lead to pregnancy-related adverse effects.

For viremia, in the HEV-NP group, one (33%) of the three viremic rabbits at 28 dpi tested positive for HEV RNA in the spleen, but all three were negative in the ovary. As for the HEV-P group, four (67%) of the six viremic rabbits at 28 dpi tested positive for HEV RNA in samples of both spleen and ovary tissues. These results indicate that viremia has a positive correlation with extrahepatic virus dissemination and that pregnancy is a risk factor.

### Elevation of serum levels of total bilirubin in HEV-3ra-infected pregnant rabbits compared to infected nonpregnant rabbits.

**FIG 5:**
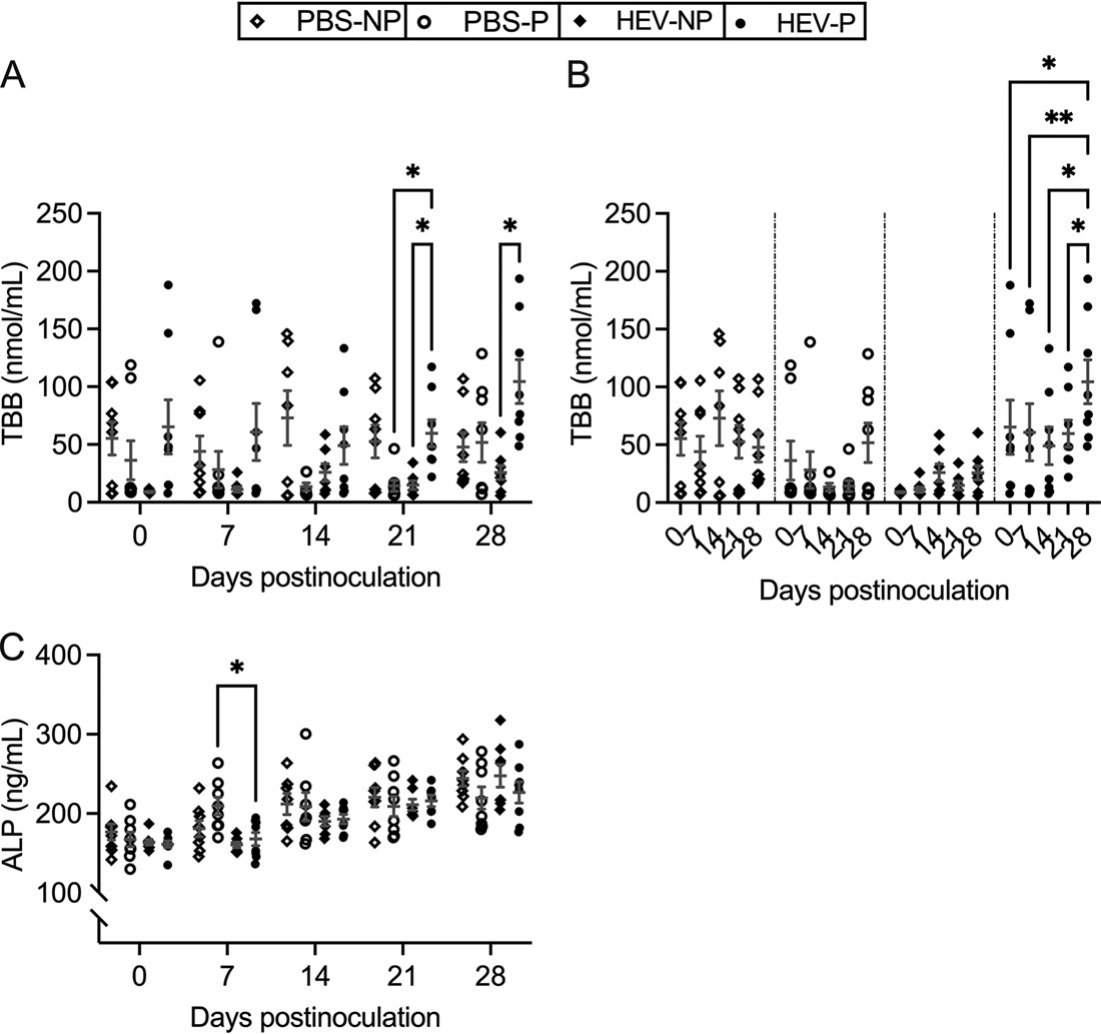
Liver function biomarkers in HEV-3ra-infected and mock-infected pregnant and nonpregnant rabbits. Serum total bilirubin (TBB) and serum liver enzyme alkaline phosphatase (ALP) were tested, using commercial rabbit-specific ELISA kits, in sera collected at 0, 7, 14, 21, and 28 dpi (necropsy, 2 to 3 days postdelivery) from HEV-3ra-infected pregnant (HEV-P) and nonpregnant (HEV-NP) rabbits and mock (PBS)-infected pregnant (PBS-P) and nonpregnant (PBS-NP) rabbits. (A) TBB concentrations for the different groups at each time point are shown. (B) TBB concentrations within each group at the different time points during the course of infection within each group are shown. (C) ALP concentrations for the different groups at each time point are shown. Data are presented as scatterplots, with each symbol indicating an individual rabbit for each time point. Bars indicate mean ± SEM. Asterisks indicate statistical significance as determined by two-way ANOVA, followed by Tukey's multiple-comparison test (*, *P < *0.05; **, *P < *0.01). PBS-NP, uninfected nonpregnant rabbits (*n* = 8); PBS-P, uninfected pregnant rabbits (*n* = 8); HEV-NP, HEV-infected nonpregnant rabbits (*n* = 8); HEV-P, HEV-infected pregnant rabbits (*n* = 8).

We assayed the concentrations of total bilirubin (TBB) and liver enzyme alkaline phosphatase (ALP) in weekly serum samples from all animals using commercial enzyme-linked immunosorbent assay (ELISA) kits. We found that HEV-P rabbits had statistically significantly higher levels of TBB at 21 dpi and 28 dpi than those in HEV-NP rabbits (*P < *0.05) (Fig. 5A). The serum levels of TBB in HEV-P rabbits were also higher at 21 dpi (*P < *0.05) than those in PBS-P rabbits (Fig. 5A). When analyzing the levels of TBB independently within each treatment group from 0 to 28 dpi, we found that only HEV-P rabbits had a significant elevation of serum TBB level at 28 dpi compared to the 0-, 7-, 14-, and 21-dpi time points (*P < *0.05 and *P < *0.01) (Fig. 5B). PBS-P rabbits also had elevated levels of serum TBB at 28 dpi but were not significantly different from those at earlier time points. Serum ALP levels were generally similar in control nonpregnant (PBS-NP), PBS-P, HEV-NP, and HEV-P rabbits, except that at 7 dpi, HEV-P rabbits had significantly lower levels than those of PBS-P rabbits (*P < *0.05) (Fig. 5C). In all four groups of rabbits, serum ALP levels increased gradually and significantly from 0 to 28 dpi, regardless of the pregnancy or infection status (data not shown).

Since our pilot rabbit studies showed that there was no change in serum levels of the liver enzyme γ-glutamyl transferase (GGT) in HEV-infected pregnant and nonpregnant rabbits at 0 to 28 dpi (Fig. S2), we decided not to assay serum GGT in the study, as no difference was expected. We also attempted to assess the levels of serum liver enzymes aspartate transaminase (AST) and alanine aminotransferase (ALT) but were unsuccessful due to technical issues with the available commercial kits for rabbits in the United States.

10.1128/mbio.00418-23.2 FIG S2 Pilot animal infection studies in pregnant and nonpregnant rabbits. To optimize the HEV infection study in pregnant rabbits and identify the appropriate virus strain, infection timing, and laboratory test parameters, we first conducted three pilot animal studies with a very small number of animals. The serum levels of γ-glutamyl transferase (GGT) in pregnant and nonpregnant rabbits experimentally infected with three different strains of genotype 3 rabbit HEV (RC-39, USRab-14, and LR) were tested with a rabbit GGT ELISA kit (MyBioSource; catalog no. MBS1601532). Results from pilot study 2 (0 to 28 dpi) and pilot study 3 (0 to 22 dpi) are shown here. There was no difference in serum level of GGT between infected and control and between pregnant rabbits and nonpregnant rabbits. Therefore, based on the pilot study result, GGT was not tested in this study. Also, based on collective virology and clinical data from the pilot studies, we chose to use HEV-3ra strain RC-39 for this study. P, pregnant rabbits; NP, nonpregnant rabbits. Download FIG S2, PDF file, 0.2 MB. Copyright © 2023 Mahsoub et al. 2023 Mahsoub et al. https://creativecommons.org/licenses/by/4.0/ This content is distributed under the terms of the Creative Commons Attribution 4.0 International license.

### The circulating level of proinflammatory cytokine IFN-γ is elevated in HEV-3ra-infected rabbits regardless of pregnancy status.

To determine the host systemic immune responses induced by HEV infection during pregnancy, we measured the weekly levels of proinflammatory cytokines interferon gamma (IFN-γ) and interleukin 6 (IL-6), as well as Th1 cytokines IL-2 and IL-12 in serum samples of both pregnant and nonpregnant rabbits using commercial ELISA kits. The cytokine levels among the four rabbit groups (PBS-NP, PBS-P, HEV-NP, HEV-P) were compared to determine the effects of pregnancy and HEV infection statuses on these cytokine responses. We found that the circulating levels of proinflammatory cytokines IFN-γ and IL-6, and Th1 cytokines IL-2 and IL-12, are similarly altered in HEV-3ra-infected rabbits regardless of pregnancy status.

**FIG 6:**
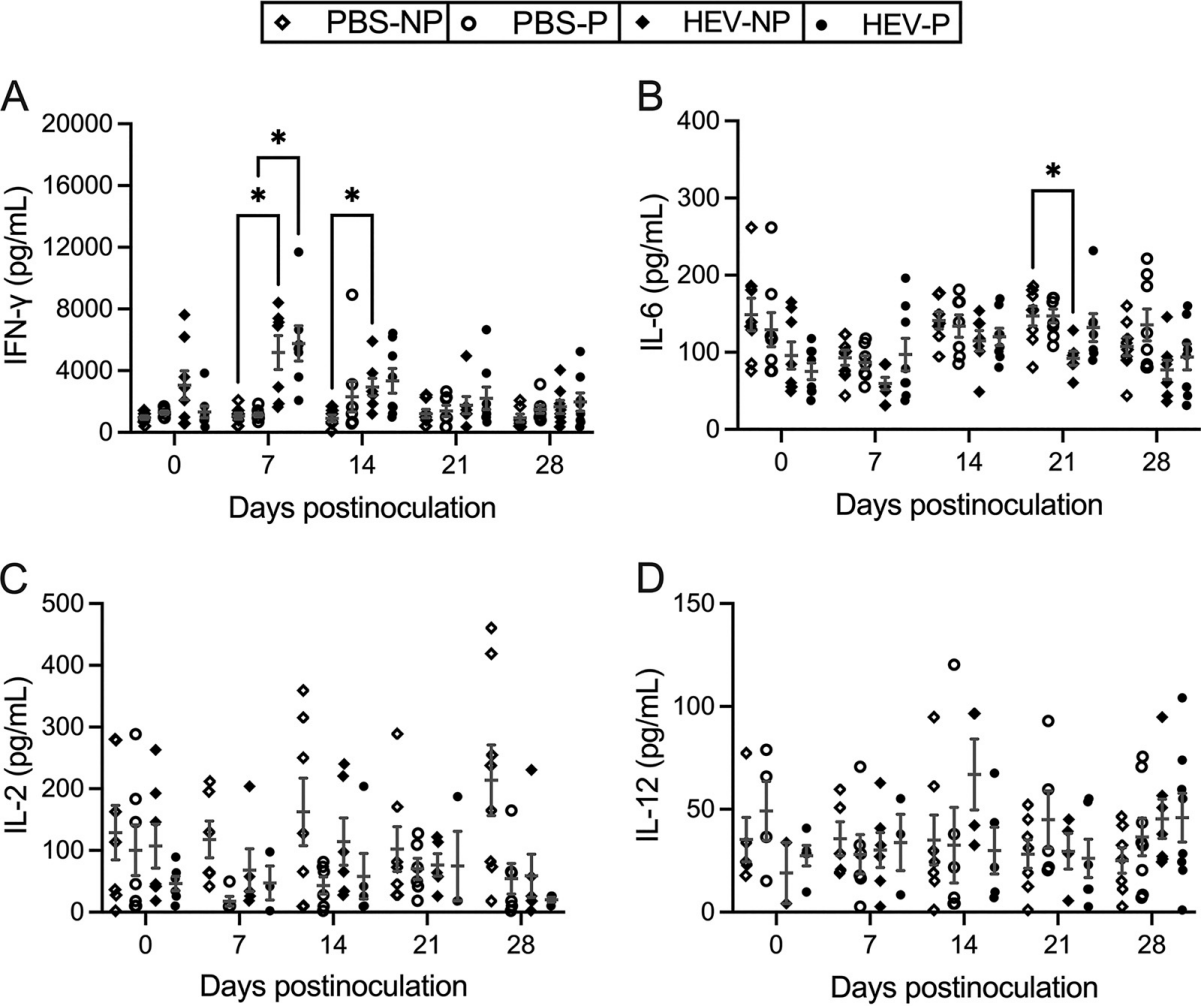
Circulating levels of selected secreted cytokines during HEV-3ra infection in pregnant and nonpregnant rabbits. The concentrations of IFN-γ (A), IL-6 (B), IL-2 (C), and IL-12 (D) in each serum sample collected at 0, 7, 14, 21, and 28 dpi (necropsy, 2 to 3 days postdelivery) from HEV-3ra-infected pregnant and nonpregnant rabbits and mock-infected (PBS) pregnant and nonpregnant rabbits. The cytokines were assayed using commercially available rabbit-specific ELISA kits, and data are presented as scatterplots, with each symbol indicating an individual rabbit at each time point. Bars indicate mean ± SEM. Asterisks indicate statistical significance as determined by two-way ANOVA, followed by Tukey's multiple-comparison test (*, *P < *0.05). PBS-NP, uninfected nonpregnant rabbits (*n* = 8); PBS-P, uninfected pregnant rabbits (*n* = 8); HEV-NP, HEV-infected nonpregnant rabbits (*n* = 8); HEV-P, HEV-infected pregnant rabbits (*n* = 8).

The levels of serum IFN-γ were significantly higher in both HEV-infected pregnant and nonpregnant rabbits throughout the course of infection. HEV-P rabbits had significantly higher levels of serum IFN-γ than PBS-P rabbits at 7 dpi (*P < *0.05), while HEV-NP rabbits had significantly higher levels than PBS-NP rabbits at 7 and 14 dpi (*P < *0.05) (Fig. 6A). At all tested time points, the HEV-infected rabbits had numerically higher serum IFN-γ levels than uninfected rabbits; the HEV-NP group was 0.77-fold to 3.88-fold (i.e., 77 to 388%) higher than the PBS-NP group, and the HEV-P group was 0.32-fold to 4.09-fold higher than PBS-P group. HEV-P rabbits had slightly higher (11 to 26%) serum IFN-γ levels than those in the HEV-NP group (Fig. 6A). The average serum IL-6 levels were generally higher in HEV-P rabbits than those in HEV-NP rabbits throughout the postinfection period, reaching 0.63-fold and 0.43-fold higher at 7 dpi and 21 dpi, respectively; however, these differences were not statistically significant. Compared to the uninfected rabbits, HEV-infected pregnant and nonpregnant rabbits had numerically lower levels of serum IL-6 from 14 to 28 dpi and from 7 to 28 dpi, respectively, with the HEV-NP group showing significantly lower levels at 21 dpi (*P < *0.05) than the PBS-NP group (Fig. 6B). At 7 dpi only, HEV-P rabbits had higher levels of serum IL-6 than PBS-P rabbits.

The average serum levels of Th1 cytokines (IL-2 and IL-12) at 0 to 28 dpi were not significantly different among the four groups, regardless of infection or pregnancy statuses (Fig. 6C and D). This was likely due to the wide individual variability among rabbits and the undetectable cytokine levels in some serum samples within each group. The average serum IL-2 levels in HEV-NP rabbits were 0.25-fold to 0.72-fold less than those in the PBS-NP group at various time points postinoculation. As for the HEV-P rabbits, serum IL-2 levels were 1.66-fold and 0.35-fold higher than those in PBS-P rabbits at 7 and 14 dpi but were 0.63-fold less at 28 dpi (2 to 3 days postdelivery). Regardless of the HEV infection status, pregnant rabbits had much lower levels of serum IL-2 than rabbits throughout the pregnancy period; PBS-P rabbits were 0.22-fold to 0.75-fold less in serum IL-2 levels, and HEV-P rabbits were 0.03-fold to 0.66-fold less than their nonpregnant counterparts.

### Upregulation in liver gene expression of IFN-γ and TNF-α in both HEV-infected pregnant and nonpregnant rabbits, with unique upregulation of IL-6 in nonpregnant, and of IL-8 in pregnant, rabbits.

To understand the host inflammatory responses to HEV infection in the liver microenvironment during pregnancy, we quantified the mRNA expression levels of a panel of proinflammatory and anti-inflammatory cytokines in the liver tissues collected at necropsy at 28 dpi from both pregnant and nonpregnant rabbits, uninfected or infected with HEV-3ra. As indicated for other parameters described above, in this study, we mainly focused on comparing the expression levels of cytokine genes in the HEV-P group with those in the HEV-NP group to examine the pregnancy factor and with those in the PBS-P group to examine the HEV infection factor.

**FIG 7:**
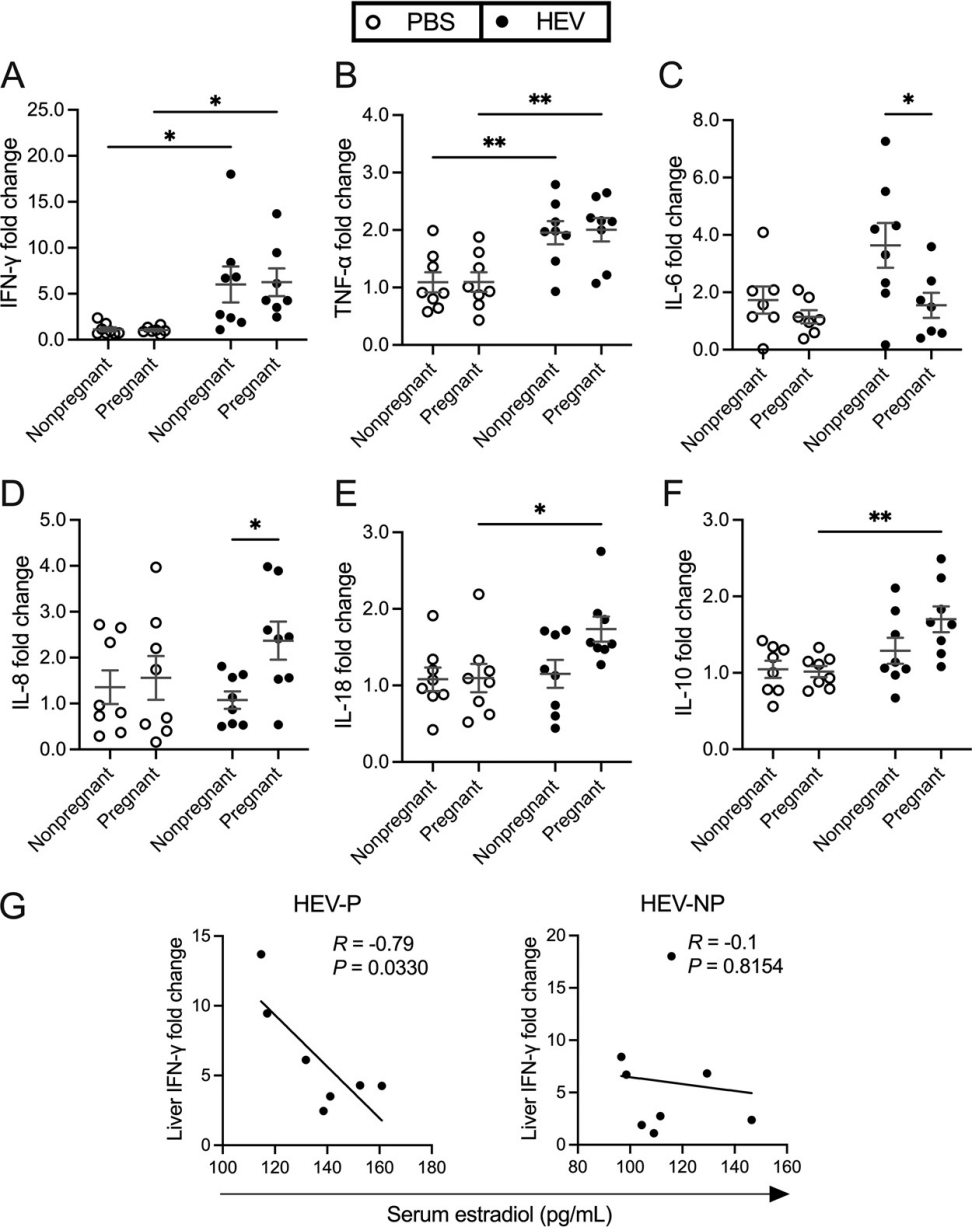
Altered gene expression levels of selected cytokines in liver tissues of HEV-3ra-infected pregnant and nonpregnant rabbits. (A to F) Relative mRNA expression of selected cytokine genes in liver tissues at necropsy at 28 dpi (2 to 3 days postdelivery) from HEV-3ra-infected nonpregnant and pregnant rabbits, as well as uninfected (PBS) pregnant and nonpregnant rabbits. RPS18 was used as a housekeeping gene control, and fold changes in the expression of target cytokine genes were calculated using the 2^−ΔΔ^*^CT^* formula. Data are presented as scatterplots, with each symbol indicating an individual rabbit. Bars indicate mean ± SEM. Asterisks indicate statistical significance as determined by two-way ANOVA, followed by Tukey’s multiple-comparison test (*, *P < *0.05; **, *P < *0.01). Each of the four groups had 7 to 8 tested rabbits. (G) Statistical correlation between serum estradiol levels at 28 dpi and liver mRNA expression levels of IFN-γ in HEV-infected pregnant and nonpregnant rabbits was determined. The Pearson’s correlation test *P* value and *R* coefficient are indicated in each graph.

The mRNA expression levels of IFN-γ and tumor necrosis factor alpha (TNF-α) (Fig. 7A and B) in the livers of both HEV-infected rabbit groups were significantly higher than those in the matching mock-infected groups (*P < *0.05 for IFN-γ and *P* < 0.01 for TNF-α), irrespective of pregnancy status. The mRNA expression level of the IL-6 gene in the liver was higher in HEV-NP rabbits than in the other groups (Fig. 7C), but it was only significant (*P < *0.05) compared to HEV-P rabbits, where IL-6 expression was similar to the PBS controls. The IL-8 gene expression in the liver was higher in HEV-P rabbits than in the other groups (Fig. 7D), but it was only significant (*P < *0.05) compared to that of the HEV-NP rabbits. There was also an upregulation in mRNA expression of IL-18 and IL-10 genes in the liver of HEV-P rabbits compared to the other groups, but the differences were only significant compared to the PBS-P group (*P < *0.05 and *P < *0.01) (Fig. 7E and F, respectively). The mRNA expression levels of IL-10 and IL-18 in the livers of HEV-P rabbits were 0.67-fold and 0.58-fold higher and 0.32-fold and 0.51-fold higher than those in the PBS-P and HEV-NP rabbits, respectively. HEV also induced an upregulation of IL-1β gene expression, which was 0.34-fold and 0.41-fold higher in HEV-P and HEV-NP rabbits than the corresponding mock-infected rabbits, though this increase was not statistically significant. Additionally, IL-2 and IL-4 gene expression levels in the livers of HEV-P rabbits were 0.28-fold and 0.27-fold higher than those in PBS-P rabbits and 0.46-fold and 0.26-fold higher than those in HEV-NP rabbits, respectively (Table S4). The differences in relative expressions of the cytokine genes tested in the study among the HEV-infected and mock-infected pregnant and nonpregnant rabbits in liver tissues are summarized in Table S4. There are wide individual variations within control and infected rabbit groups. Interestingly, we found that the average serum level of estradiol (E2) in HEV-P rabbits at 28 dpi had a significant negative correlation (*r* = −0.79, *P = *0.0330) with liver IFN-γ mRNA levels compared to HEV-NP rabbits, which showed no such linear correlation (Fig. 7G).

10.1128/mbio.00418-23.6 TABLE S4 Differences in fold change of gene expression levels of selected cytokines, chemokines, and pregnancy-related genes in the liver tissues among the four study groups of pregnant and nonpregnant female rabbits experimentally infected with HEV-3ra or mock-infected with PBS. Download Table S4, DOCX file, 0.02 MB. Copyright © 2023 Mahsoub et al. 2023 Mahsoub et al. https://creativecommons.org/licenses/by/4.0/ This content is distributed under the terms of the Creative Commons Attribution 4.0 International license.

In addition to the cytokine genes, the mRNA expression levels of the pregnancy-related genes, estrogen receptors (*ESR1* and *ESR2*), progesterone receptors (*PRMC1* and *PRMC2*), and progesterone-induced blocking factor (*PIBF-1*), were also quantified in the liver and placenta tissues collected at necropsy at 28 dpi. No significant changes in these gene expressions were found between HEV-infected or uninfected pregnant or nonpregnant rabbits (Table S4).

### Pronounced histological lesions in spleens of HEV-3ra-infected pregnant rabbits, with similar liver lesions in infected pregnant and nonpregnant rabbits.

**FIG 8:**
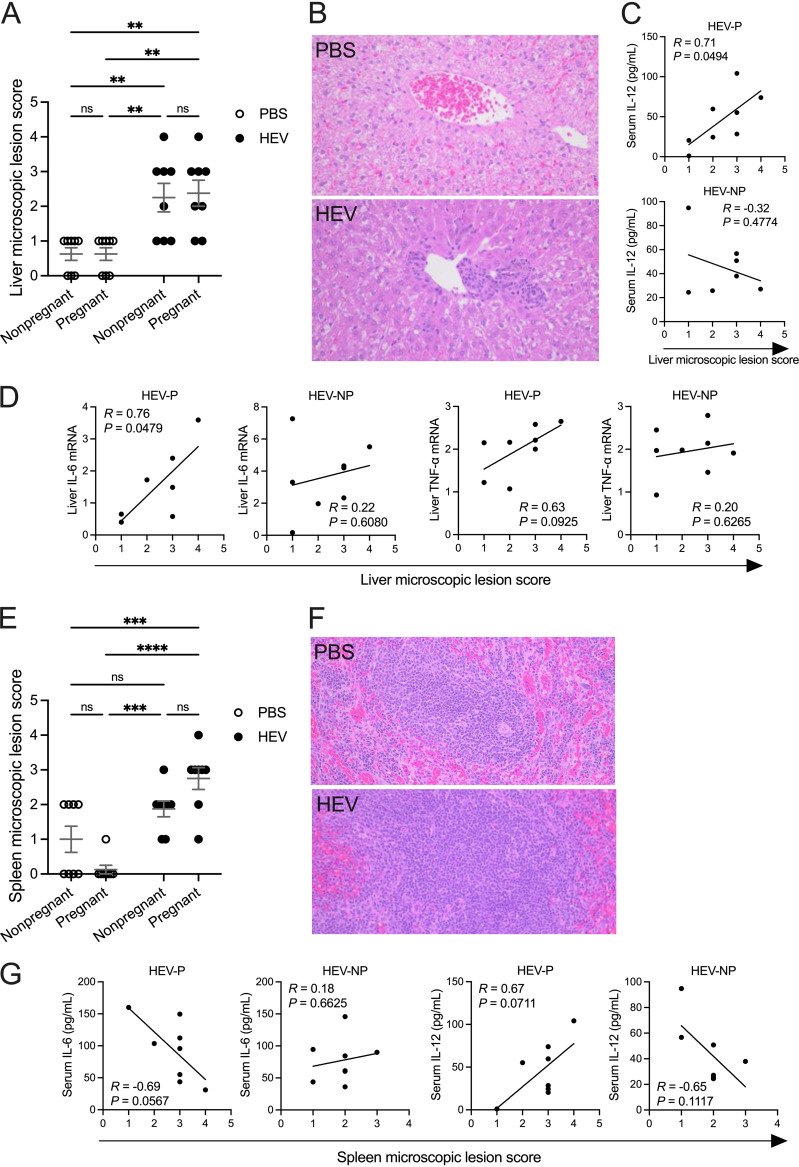
Histological lesions in liver and spleen of HEV-3ra-infected pregnant and nonpregnant rabbits. (A) Histological lesion scores of livers at necropsy at 28 dpi (2 to 3 days postdelivery) from the indicated groups. (B) Representative micrographs of liver sections in mock-infected and HEV-3ra-infected rabbits. (C and D) Statistical correlation between liver lesion scores and serum levels of IL-12 at 28 dpi or liver lesion scores and liver mRNA expression levels of IL-6 and TNF-α genes, respectively, in HEV-3ra-infected pregnant and nonpregnant rabbits. The Pearson’s correlation test *P* value and *R* coefficient are indicated in each graph. (E) Histological lesion scores of spleens at necropsy at 28 dpi (2 to 3 days postdelivery) from the indicated groups. (F) Representative micrographs of spleen sections in mock-infected and HEV-3ra-infected rabbits. (G) Statistical correlation between spleen lesion scores and serum levels of IL-6 and IL-12 at 28 dpi, respectively, in HEV-3ra-infected pregnant and nonpregnant rabbits. The Pearson’s correlation test *P* value and *R* coefficient are indicated in each graph. Histopathology data are presented as scatterplots, with each symbol indicating the score from an individual rabbit. Bars indicate mean ± SEM. Asterisks indicate statistical significance as determined by two-way ANOVA, followed by Tukey’s multiple-comparison test (**, *P < *0.01; ****, *P < *0.0001). In each group, *n* = 8 rabbits.

At necropsy at 28 dpi, a panel of tissue samples, including liver and spleen, was collected and processed for histological examinations. The livers of the HEV-infected rabbits had histological lesions characterized by phlebitis and intralobular inflammation (Fig. 8B). The average score of liver histological lesions was not significantly different between HEV-infected pregnant and nonpregnant rabbits; however, the scores in both groups were significantly higher (*P = *0.0021 and 0.0044, respectively) than those in their matching uninfected rabbit groups (Fig. 8A). The spleens showed granular cell necrosis at various levels in the different groups, but histological lesions were most severe in HEV-P rabbits (Fig. 8F). The average score of splenic histological lesions in HEV-infected pregnant rabbits was significantly higher (*P < *0.0001) than that in uninfected pregnant rabbits and numerically higher than that in HEV-infected nonpregnant rabbits. Splenic lesion scores were not different between HEV-infected and uninfected nonpregnant rabbits (Fig. 8E). No significant histological changes were observed in kidney, ovary, or placenta tissues from infected or uninfected rabbits. Also, since data from our pilot animal studies showed no histological changes in the small intestinal tissues of HEV-infected rabbits, we did not examine these tissues in the study. There was no direct correlation between viral loads and histologic lesion scores in the liver of both pregnant and nonpregnant rabbits (data not shown). However, in HEV-P rabbits, there were positive linear correlations between liver lesion scores and liver mRNA levels of IL-6 (*r* = 0.76, *P = *0.0479) and TNF-α (*r* = 0.63, *P = *0.0925) (Fig. 8D) and serum IL-12 levels at 28 dpi (*r* = 0.71, *P = *0.0494) (Fig. 8C). Also, in HEV-P rabbits, we found that spleen lesion scores correlated positively (*r* = 0.67, *P = *0.0711) with serum IL-12 levels at 28 dpi and negatively (*r* = −0.69, *P = *0.0567) with serum IL-6 levels (Fig. 8G). On the contrary, in HEV-NP rabbits, only a moderate negative correlation (*r* = −0.65, *P = *0.1117) was observed between spleen lesion scores and serum IL-12 levels at 28 dpi; other correlations for liver and spleen lesions were insignificant (Fig. 8C, D, and G).

### Pregnancy-associated sex hormone estradiol, but not estrone, correlates positively with high viral loads in pregnant rabbits.

At 7 days of gestation, i.e., the seventh day after copulation, rabbits in the two pregnant groups were inoculated with HEV or PBS (see Materials and Methods), and this was the 0-dpi time point. The levels of pregnancy hormones (estrone [E1] and estradiol) were measured in the serum samples of all pregnant and nonpregnant rabbits at 0, 7, 14, 21, and 28 dpi (i.e., 2 to 3 days postdelivery when necropsy was performed).

**FIG 9:**
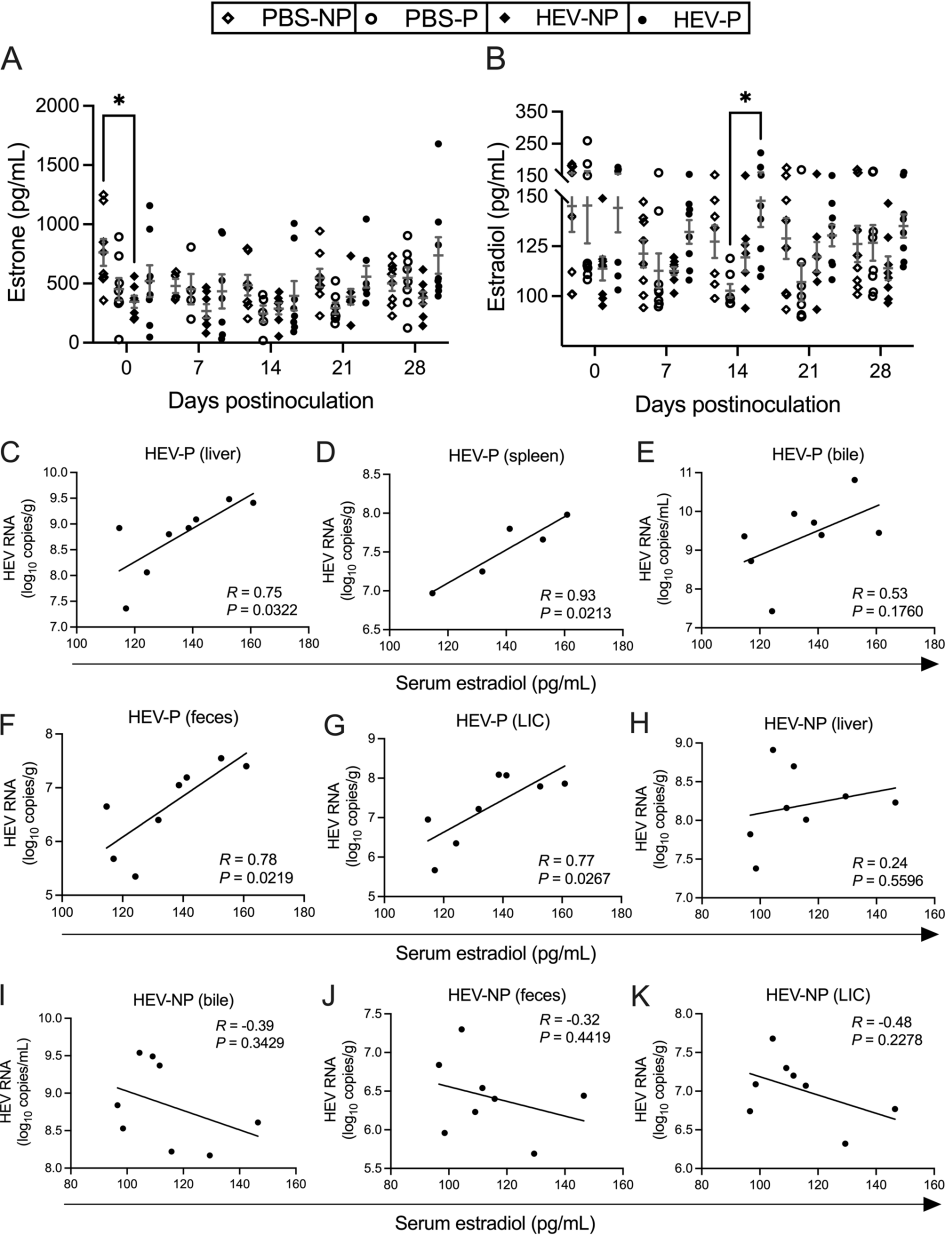
Serum levels of pregnancy-associated sex hormones (estrone and estradiol) in HEV-3ra-infected or mock-infected pregnant and nonpregnant rabbits and their correlations with viral loads in fecal, bile, liver, and spleen tissues. The concentrations of estrone (A) and estradiol (B) in serum samples collected at 0, 7, 14, 21, and 28 dpi (necropsy, 2 to 3 days postdelivery) from HEV-3ra-infected or mock (PBS)-infected pregnant and nonpregnant rabbits. The hormones were quantified using commercially available rabbit-specific ELISA kits, and the data are presented as scatterplots with each symbol indicating an individual rabbit for each time point. Bars indicate mean ± SEM. Statistical significance was determined by two-way ANOVA, followed by Tukey's multiple-comparison test (*, *P < *0.05). PBS-NP, uninfected nonpregnant rabbits (*n* = 8); PBS-P, uninfected pregnant rabbits (*n* = 8); HEV-NP, HEV-infected nonpregnant rabbits (*n* = 8); HEV-P, HEV-infected pregnant rabbits (*n* = 8). Statistical correlation between serum levels of estradiol at 28 dpi and viral RNA loads in liver, fecal samples, large intestinal contents (LIC), and spleen at 28 dpi were determined for HEV-infected pregnant (C to G) and HEV-infected nonpregnant (H to K) rabbits. The Pearson’s correlation test *P* value and *R* coefficient are indicated in each graph. No spleen correlation was determined for HEV-NP, as only one spleen sample tested positive.

The average serum E1 levels did not differ significantly among all treatment groups at the various days postinoculation. Nevertheless, at 14 to 28 dpi, the average concentrations of E1 in HEV-P rabbits were notably higher (0.34-fold to 0.91-fold) than those in PBS-P rabbits. The average E1 levels in HEV-P rabbits were also higher (0.38-fold to 0.95-fold) than those in HEV-NP rabbits, while no such differences were observed between the PBS-NP and PBS-P groups (Fig. 9A). The average serum levels of E2 in HEV-P rabbits were significantly higher (0.44-fold) than those in PBS-P rabbits at 14 dpi (*P < *0.05). The average serum E2 levels in HEV-P rabbits were slightly higher than those in HEV-NP rabbits, while PBS-P rabbits had lower serum E2 levels than those in PBS-NP rabbits (Fig. 9B). Collectively, these results indicate that the serum levels of pregnancy hormones, E1 and E2, in HEV-infected rabbits follow different kinetics from those in uninfected rabbits.

We examined the correlations between serum E1 and E2 levels in HEV-infected pregnant and nonpregnant rabbits and the corresponding viral loads in serum, feces, bile, and tissue samples. We found that there are statistically significant, positive linear correlations between the serum levels of E2 at 28 dpi in HEV-P rabbits and viral loads in liver (*r* = 0.75, *P = *0.0322) (Fig. 9C), spleen (*r* = 0.93, *P* = 0.0213) (Fig. 9D), fecal samples at 28 dpi (*r* = 0.78, *P = *0.0219) (Fig. 9F), and LIC (*r* = 0.77, *P* = 0.0267) (Fig. 9G); a positive correlation was also observed with viral loads in bile but was not statistically significant (*r* = 0.53, *P = *0.1760) (Fig. 9E). In the HEV-NP group, there was no significant correlation between the serum levels of E2 at 28 dpi and viral loads in liver, bile, fecal, or LIC samples at 28 dpi (Fig. 9H to K). The correlations between E2 and viral loads in serum, spleen, and ovary samples were not determined for HEV-NP rabbits, as there are not enough positive samples for this group. Additionally, there is no significant correlation between serum E1 levels at 28 dpi and viral loads in samples collected from HEV-infected pregnant and nonpregnant rabbits (data not shown).

## DISCUSSION

HEV-1 infection in pregnant women is associated with severe clinical outcomes, including FHF, stillbirth, miscarriage, and maternal and fetal mortality (2, 15–17, 59). Possible factors that are thought to contribute to HEV-1-associated severe disease during pregnancy include host immunity, levels of secreted cytokines, pregnancy hormones, and other host and viral factors (55). The underlying mechanisms that contribute to adverse clinical outcomes in pregnant women have not been fully investigated, in part due to the absence of a suitable pregnant animal model for HEV infection. Attempts to reproduce HEV-1-associated severe diseases in pregnant rhesus macaques infected with HEV-1 (23) or in pregnant sows infected with HEV-3 (24) have not been successful. Recently, an *ex vivo* model utilizing the decidua basalis and fetal placenta was used to compare HEV-1- and HEV-3-induced pathogenic effects at the maternal-fetal interface. It was reported that HEV-1 replicated at significantly higher levels than HEV-3, causing dysregulation in the secretion of several cytokines, which contributed to severe decidual and placental tissue injuries (60). Although this *ex vivo* study provided valuable information regarding the potential mechanism of HEV-1-induced pregnancy complications, a relevant animal model system is needed to more definitively investigate the mechanisms of HEV-induced liver damage and pregnancy adverse events.

Pregnant rabbits have evolved as a potential model for HEV-induced adverse clinical outcomes observed in a high percentage of HEV-infected pregnant women (52–54), although HEV-1-associated fulminant hepatitis in pregnant women (61–63) has not been observed in the rabbit studies. In two studies from the same research group in China, infected pregnant rabbits showed different incidences of adverse pregnancy outcomes, including miscarriage (up to 33%) and stillbirth (up to 50%), as well as severe liver disease and maternal death, which happened several weeks postdelivery (52, 54). In another study from Korea, infected pregnant rabbits reportedly had 67 to 80% fetal mortality, with mild to moderate lymphocytic hepatitis in liver tissues at necropsy at 1 to 2 days postdelivery (53). However, an in-depth investigation into the underlying molecular mechanism(s) of HEV-associated severe adverse clinical outcomes during pregnancy, which remains unknown, was not conducted.

Since two groups now reported HEV-induced severe diseases in pregnant rabbits, and since rabbit is a more convenient model than pig and rhesus monkey because of the large litter size (average, 8 kits per litter) and shorter gestation period (average, 32 days), in this present study, we aimed to utilize the pregnant rabbit model to systematically determine the roles and underlying mechanisms of pregnancy-associated sex hormone (estrogens), host immune responses, and viral replication kinetics in liver and extrahepatic tissues in the development of HEV-induced adverse clinical outcomes associated with pregnancy. The results from this study showed that HEV-3ra-infected rabbits had a significantly lower number of kits per litter than uninfected pregnant rabbits, with a fetal loss of ~23%; however, we were unable to reproduce the other pregnancy-associated severe outcomes such as maternal death or stillbirth reported by the other two groups (52, 53). The discrepancy may be attributed to experimental conditions, differences in the virus strains used, or rabbit strain susceptibility since virus strain and host susceptibility are two of the determining factors of HEV infection outcome (55). The inability to reproduce other severe clinical outcomes of HEV infection during pregnancy (except for fetal loss) in HEV-3ra-infected pregnant rabbits also suggests virus genotype-specific pathogenicity since the reported severe clinical outcomes during pregnancy are almost all associated with HEV-1 infection in humans. It is worth noting that the high maternal mortality observed in one of the reported previous studies (52) occurred 6 to 7 weeks postdelivery, while in the other study (54), only one pregnant rabbit out of six died on the 34th day of pregnancy. It is likely that the fetal loss observed in the present study has occurred at an early point during pregnancy when the soft fetal tissues and bones were retained and absorbed in the body with no clinical complications. Dead fetuses in rabbits can be completely resorbed if the death occurred before day 21 of gestation (64).

In this study, we systematically quantified and compared viral loads in liver as well as in extrahepatic tissues, including serum, feces, intestinal contents, spleen, kidney, and ovary tissues in both HEV-infected pregnant and nonpregnant rabbits during the course of HEV-3ra infection. We found that viral loads in fecal samples at all time points and in LIC at necropsy were generally higher in infected pregnant rabbits than in nonpregnant rabbits, thus indicating an enhanced viral replication in pregnant rabbits. Furthermore, the onset of viremia was 1 week earlier with higher incidence and longer duration in HEV-infected pregnant rabbits than in infected nonpregnant rabbits. Importantly, we found that incidence of viral dissemination and viral loads in tissues and bile were either significantly or numerically higher in infected pregnant rabbits than in nonpregnant rabbits. Intriguingly, our results of viral loads, viremia incidence, and virus dissemination in HEV-infected pregnant rabbits are similar to what has been reported in an immunosuppressed rabbit model (58). Since we had to euthanize rabbits shortly after delivery, at 28 dpi, we were unable to explore the differences between pregnant and nonpregnant rabbits in fecal virus shedding, viremia, and virus clearance for a longer period of time. It is worth noting that the only pregnant rabbit (rabbit 33) that seroconverted by 28 dpi with a high level of anti-HEV IgG antibodies (see Fig. S1 in the supplemental material) did not have viremia at any time point during the course of HEV infection. This rabbit also had very low viral loads in fecal and bile samples, the lowest viral loads in LIC and liver tissues, and no viral RNA in spleen or placenta. Moreover, this rabbit delivered the highest number of kits per litter (*n* = 9) compared to the other HEV-infected pregnant rabbits in the HEV-P group, implying a much lower burden of virus infection. Data from this individual rabbit may signify the importance of negative impact on clinical outcomes of enhanced virus replication during pregnancy and the importance of efficient antibody response to prevent viremia in pregnant rabbits and thus prevent severe diseases.

10.1128/mbio.00418-23.1 FIG S1 Anti-HEV IgG antibody responses in HEV-3ra-inoculated or mock-inoculated pregnant and nonpregnant female rabbits. Individual values of optical density at 450 nm (OD_405_) (8 rabbits per group) and cutoff values (dashed line) are plotted. (A) PBS-NP, PBS-inoculated nonpregnant rabbits; (B) PBS-P, PBS-inoculated pregnant rabbits; (C) HEV-NP, HEV-inoculated nonpregnant rabbits; (D) HEV-P, HEV-inoculated pregnant rabbits. Among the four groups, only two rabbits (rabbits 31 and 33) in the HEV-infected pregnant rabbit group (D) tested seropositive for anti-HEV IgG antibodies at 28 dpi. Rabbit 31 had an OD value that is slightly higher than the cutoff value, while rabbit 33 had a very high OD value above the cutoff. The lack of seroconversion in most of the infected rabbits is expected since the experiment lasted only 28 days. Anti-rabbit HEV IgG antibodies titers were assayed using an HEV ORF2 antigen-based ELISA. Briefly, serum samples were collected weekly from 0 to 28 days postinoculation (dpi). Serum samples were diluted at 1:200 in blocking buffer. Preimmune and hyperimmune anti-HEV positive rabbit sera were included as negative and positive controls, respectively. Download FIG S1, PDF file, 0.2 MB. Copyright © 2023 Mahsoub et al. 2023 Mahsoub et al. https://creativecommons.org/licenses/by/4.0/ This content is distributed under the terms of the Creative Commons Attribution 4.0 International license.

Estrogen is important for the maintenance of pregnancy (65, 66). In this study, we found that the serum levels of estrone and estradiol were generally higher in HEV-infected pregnant rabbits than in uninfected pregnant rabbits at different time points post-inoculation therefore indicating a possible role of HEV infection in regulating the estrogen levels. A study in pregnant women showed significantly higher levels of estrogen, progesterone, and β-human chorionic gonadotropin in HEV-positive FHF patients than in HEV-negative FHF patients (59). An *in vitro* study reported that HEV infection regulates the estrogen-signaling pathway (67). In this present study, we found strong positive correlations between the serum levels of estradiol at 28 dpi in HEV-infected pregnant rabbits and higher viral loads in liver, spleen, bile, and fecal samples at 28 dpi, as well as intestinal contents. We also observed a significant positive correlation between serum estradiol levels and IFN-γ gene expression level in the liver of infected pregnant rabbits, suggesting that estradiol in pregnant women may suppress the intrahepatic IFN-mediated antiviral activity, leading to enhanced viral replication observed in this study. These results suggest that estradiol may play a role in promoting viral replication, thereby leading to extrahepatic viral dissemination in infected pregnant rabbits. In *in vitro* studies, estrogen was reported to either enhance (68) or have no effect on HEV replication (69) in cultured hepatocellular carcinoma cells Huh7, signifying the need for optimizing HEV culture systems to study hormonal effects on HEV replication. Progesterone was reported to enhance HEV replication in human liver cells (70); however, we were unable to assess the potential role of progesterone in mediating HEV infection in pregnant rabbits due to technical issues with reagents to measure rabbit progesterone.

Pregnancy can alter maternal immune responses to allow the allogeneic fetus to survive, but this may also predispose the pregnant mother to infections and severe clinical consequences (71–75). Since we found in this study that fetal loss occurred during early stages of pregnancy, we quantified the serum levels of 4 different cytokines (IFN-γ, IL-6, IL-2, and IL-12) in both HEV-infected and uninfected pregnant rabbits at 7 and 14 dpi (i.e., days 14 and 21 of pregnancy) to determine whether there is a potential correlation with fetal loss. We showed that the mean serum IFN-γ levels in HEV-P rabbits were significantly higher at 7 dpi and numerically higher at 14 dpi than in PBS-P rabbits. On the other hand, in HEV-NP rabbits, serum IFN-γ levels were significantly higher at 7 and 14 dpi than those in PBS-NP rabbits, indicating that the HEV-induced IFN-γ response subsided earlier in HEV-infected pregnant rabbits than in nonpregnant rabbits. These findings suggest that the HEV-induced spike in serum IFN-γ levels during the earlier stage of virus infection may be associated with fetal loss in pregnant rabbits. Remarkably, the incidence of viremia increased substantially in HEV-infected pregnant rabbits from 2/8 rabbits at 7 to 14 dpi to 6/8 rabbits at 21 to 28 dpi, following the decline in serum IFN-γ level. HEV-infected nonpregnant rabbits, which had significantly higher serum IFN-γ levels at 7 and 14 dpi, only had a very low incidence of viremia. Overall, the serum levels of proinflammatory cytokines (higher IFN-γ and IL-6 in infected pregnant rabbits than in infected nonpregnant rabbits) during HEV infection in pregnant rabbits did not seem to have been adequately moderated, which may contribute to the observed fetal loss during early stages of HEV infection in pregnant rabbits. Interestingly, in this study, we found there is a low level of serum IL-6 in the HEV-infected groups. Studies of peripheral blood mononuclear cells (PBMCs) in HEV-infected pregnant and nonpregnant patients have shown alterations in the proportion of lymphocyte subpopulations as well as PBMC cytokine responses (76–78). IL-6 is secreted in the blood by a variety of cells, including Th (CD4-positive [CD4^+^]) cells and monocytes; therefore, reduction of the number of any of these cells may affect the overall level of IL-6 in the blood. It has been reported that HEV-positive pregnant FHF women had significantly lower CD4^+^ cell counts than HEV-negative pregnant FHF women or control patients (59). It is possible that the low level of serum IL-6 in HEV-infected rabbits observed in this study may be due to a reduction in the number of IL-6-secreting cells in peripheral blood.

Currently, there is a lack of information about the intrahepatic inflammatory responses during HEV infection in pregnant women. Therefore, in this study, we quantified the relative mRNA expression levels of a panel of proinflammatory and anti-inflammatory cytokine genes in liver tissues at necropsy (at 28 dpi or 2 to 3 days postdelivery), which had histological lesions in both HEV-infected pregnant and nonpregnant rabbits. We found that there were numerically higher expression levels of IL-18 and IL-10, significantly higher IL-8 mRNA levels, and significantly lower IL-6 mRNA levels in the liver of HEV-P rabbits than in the liver of HEV-NP rabbits. The significantly higher levels of the proinflammatory cytokine IL-18 and anti-inflammatory cytokine IL-10 in HEV-P rabbits than in PBS-P rabbits suggest that, in some infected pregnant rabbits, the host immune system may reach inflammatory homeostasis in the liver. In HEV-infected pregnant rabbits, the negative correlation of serum estradiol with IFN-γ gene expression in liver and its positive correlation with viral loads in liver may be one potential mechanism for how estrogen can promote viral replication at the site of infection. As indicated above, unlike in the HEV-NP group, the level of liver IL-6 mRNA expression in the HEV-P group was not upregulated, which may contribute to the increased viral titer (and histopathology) in the liver of pregnant rabbits. In addition to its protective role during liver injury (79), in a mouse study of hepatitis B virus, IL-6 released by Kupffer cells in the liver had antiviral activities through suppressing transcription of pregenomic RNA, antigen expression, and replication in infected hepatocytes (80). In this study, we found that IL-6 was not upregulated in the liver of HEV-P rabbits, which may be due to pregnancy-associated dysregulation or suppression of Kupffer cells’ function in the liver. High concentrations of 17β-estradiol (E2), similar to those found in pregnant women, resulted in reduced production of proinflammatory cytokines, such as IL-6, in human-derived monocytes and macrophages, while low E2 concentration enhanced the production of these cytokines (81). Since the cytokine responses at the gene expression level are usually transient, it would be more informative to determine the liver cytokine responses to HEV infection at multiple time points postinfection to assess the dynamics of the liver inflammatory status at the different infection stages. Studies showed that IFN-γ, TNF-α, IL-6, IL-8, and IL-10 are among the most important cytokines that contribute to the inflammatory response to rabbit hemorrhagic disease virus, which also targets the liver (82, 83). Overall, the findings in this study on systemic and hepatic cytokine responses suggest that the fetal loss and development of severe liver disease during HEV infection in pregnant women may occur through two distinguishable, but overlapping, immunological pathways and time frames.

In this study, regardless of pregnancy status, we found liver damage in both HEV-infected groups during necropsy at 28 dpi and prior to the development of detectable serum anti-HEV IgG antibodies, with a median histological lesion score of 2.5 (2 corresponds to 6 to 10 focal infiltrates, and 3 corresponds to 11 to 20 focal infiltrates per 10 liver lobules). Through regression analyses, we showed that liver IL-6 and TNF-α mRNA expression levels and serum IL-12 levels at 28 dpi may contribute to liver histological lesions in HEV-infected pregnant rabbits. Serum total bilirubin is a common marker of liver function, and a high level of blood TBB is indicative of liver injury (84). HEV-infected patients had elevated levels of TBB compared to uninfected ones (20). In the present study, we found that serum levels of TBB in HEV-infected pregnant rabbits were higher than those in HEV-infected nonpregnant rabbits and uninfected pregnant rabbits. This indicates that the HEV-induced liver histological lesions can produce more pronounced negative effects in pregnant animals than in nonpregnant animals. In addition to liver, we also found that spleens in HEV-infected pregnant rabbits had more pronounced histological lesions than those in infected nonpregnant rabbits, indicating that pregnancy can exaggerate HEV-associated splenic damage. Also, HEV RNA was found in high titers in the spleen of 62.5% of HEV-infected pregnant rabbits, which corroborates the splenic histological lesions. Collectively, the data from this study emphasize that, in pregnant animals, extrahepatic virus dissemination can cause extrahepatic organ damage, which may further aggravate the pregnancy-associated adverse outcome during HEV infection.

In summary, we showed that pregnant rabbits experimentally infected with HEV-3ra can recapitulate some (significant fetal loss, spleen and liver injury), but not all, pregnancy-associated adverse clinical outcomes seen in HEV-infected pregnant women. Although liver histological lesions were similar in both HEV-infected pregnant and nonpregnant rabbits, the underlying factors (higher viral loads, inflammatory cytokines, and pregnancy hormones) contributing to severe diseases are different and more pronounced in pregnant rabbits. The HEV-infected pregnant animals showed higher viral replication, worse viremia markers, higher extrahepatic virus dissemination, and altered inflammatory responses. The positive correlations between estradiol levels and both viral replication and cytokine responses in HEV-infected pregnant rabbits point out a significant role of pregnancy hormones in HEV pathogenesis during pregnancy.

## MATERIALS AND METHODS

### Preparation of HEV-3ra infectious virus stock.

HEV-3ra GDC9 (GenBank accession no. FJ906895), originally isolated from rabbits in China (37, 44), was provided by Youchun Wang (National Institutes for Food and Drug Control in Beijing, China), for a previous infection study (designated strain RC-39) in our laboratory (42). To produce a high infectious titer of an HEV-3ra RC-39 virus stock for the present study, we intravenously inoculated two specific-pathogen-free (SPF) New Zealand White (NZW) rabbits each with 3 mL fecal suspension of HEV-3ra RC-39 from a previous study (42). Daily fecal samples were collected from each rabbit and quantified for HEV-3ra RNA loads by RT-qPCR. When the infected rabbits reached the peak viral RNA titer in feces, the animals were necropsied, and bile and large intestinal contents (LIC) were collected during necropsy to prepare infectious virus stocks as 10% fecal suspensions in PBS buffer. The 10% fecal suspensions were clarified at 3,000 rpm for 15 min, filtered through a 40-μm cell strainer, and stored in aliquots at −80°C as the infectious virus stock. The absence of bacterial contamination and toxins in the virus stock was verified by ToxinSensor chromogenic LAL endotoxin assay kit (GenScript, Piscataway, NJ). The genomic RNA titer of the infectious HEV-3ra stock was determined by RT-qPCR, and the optimal infectious dose, as well as the time point for rabbit infection, were experimentally determined through pilot HEV-3ra infection studies in NZW pregnant and nonpregnant rabbits. In the pilot studies, we inoculated a very small number of pregnant rabbits with 1 to 3 mL of virus stock at 7 or 14 days of pregnancy to identify the optimal infection conditions (see Fig. S2 in the supplemental material), as described below for this study. Based on the collective virological and clinical data from the pilot animal studies, we chose to use HEV-3ra strain GDC9 (or RC-39) for this pregnant rabbit HEV infection study (Fig. S2).

### Experimental design for HEV-3ra infection in pregnant and nonpregnant rabbits.

All rabbit studies were approved by the Institutional Animal Care and Use Committee at Virginia Tech (IACUC number 19-047). A total of 32 HEV-negative, 6.5-month-old, NZW female rabbits (16 pregnant and 16 nonpregnant; purchased from Envigo, Denver, PA) were randomly divided into 4 groups of 8 rabbits per group, control nonpregnant (PBS-NP), control pregnant (PBS-P), infected nonpregnant (HEV-NP), and infected pregnant (HEV-P). Prior to arrival, serum samples were collected from all animals and tested to be free of anti-HEV IgG antibodies and HEV RNA using an ELISA and RT-qPCR or nested RT-PCR, respectively. Upon arrival, rabbits were housed separately in cages in an animal biosafety level 2 (ABSL-2) facility at Virginia Tech. At 7 days of gestation, the two HEV-3ra infection groups were intravenously inoculated with HEV-3ra infectious virus stock (~2 × 10^6^ genomic RNA copies per rabbit), while the rabbits in the two negative-control groups were each similarly mock-inoculated with PBS buffer.

Fecal and serum samples were collected and stored at −80°C from all rabbits prior to inoculation and twice a week (feces) or weekly (serum) thereafter until necropsy at 28 days postinoculation (dpi), which coincided with 2 to 3 days postdelivery for the pregnant rabbits. Pregnant rabbits were monitored daily for clinical signs, and the number of kits born (alive and dead) per litter was counted and recorded immediately after each rabbit’s delivery. When pregnant rabbits gave birth, samples of placenta tissues were collected, and during necropsy at 28 dpi, samples of liver, spleen, kidney, and ovary tissues were collected from all rabbits. One set of tissue samples was snap-frozen on dry ice and stored at −80°C for extracting RNA to quantify viral loads and various gene expression levels. A second set of tissue samples was fixed in 10% neutral buffered formalin and processed for routine histological evaluation. Bile and LIC samples were also collected at necropsy and stored at −80°C.

### Histological examination.

Tissue slides were stained with hematoxylin and eosin and examined in a blinded fashion by a board-certified veterinary pathologist (T.L.). Liver microscopic lesions were scored from 0 to 4 based on the presence of phlebitis and intralobular inflammation (0, normal; 1, 1 to 5 foci; 2, 6 to 10 foci; 3, 11 to 20 foci; 4, >20 foci per 10 liver lobules). Spleen histological lesions were scored from 0 to 4 based on the presence of granular cell necrosis (0, normal; 1, minimal; 2, mild; 3, moderate; 4, severe). Kidney, placenta, and ovary tissues were also evaluated for the presence or absence of histological lesions.

### RNA extraction.

Total RNA was extracted from 200-μL aliquots of 10% (wt/vol) fecal suspensions in PBS, 20% (wt/vol) LIC suspensions in PBS, and 50 μL of bile, using 1 mL of TRI reagent (Molecular Research Center), following the manufacturer’s instructions. Total RNA was extracted from 100 μL of serum samples using Quick-RNA viral kit (Zymo Research), following the manufacturer’s instructions. Samples of tissues (liver, spleen, placenta, ovary, and kidney) were prepared as 10% (wt/vol) tissue homogenates in TRI reagent using gentleMACS dissociator, following the “frozen tissue” RNA program instructions. Total RNAs were extracted from the respective 10% tissue homogenate of spleen, ovary, and kidney by following the TRI reagent protocol. For liver and placenta samples, after homogenization and centrifugation, the supernatants were transferred to genomic DNA (gDNA) eliminator spin columns in the RNeasy Plus minikit (Qiagen), and total RNA was subsequently extracted following the manufacturer’s instructions. The quality and concentration of extracted total RNAs from each tissue were determined using NanoDrop 1000 spectrophotometer (Thermo Scientific).

### RT-qPCR for quantification of HEV-3ra RNA.

HEV-3ra genomic RNA was quantified using SensiFAST probe No-ROX one-step kit (Bioline USA Inc.) with primers and probe listed in Table 3, following a protocol described previously (85), with some modifications. The RT-qPCR assays were performed in a CFX96 real-time PCR system, C1000 thermal cycler (Bio-Rad Laboratories). A one-step RT-qPCR thermal cycling protocol was used as follows: 45°C for 10 min and 95°C for 2 min, followed by 40 cycles of 95°C for 5 s and 60°C for 20 s. *In vitro*-transcribed HEV-3ra RNAs were used to generate a standard curve in each RT-qPCR run, which covered a quantification range from 4 × 10^2^ to 4 × 10^7^ RNA copies per reaction. The total RNA extracted from each of the tissue samples was diluted at 250 ng/μL, thus allowing the use of 1 μg total RNA (4 μL)/reaction as recommended in the kit. The PCR quantification data were then used to calculate the viral RNA loads as genome copies per gram of tissue. Fold increase in viral loads in pregnant rabbits (HEV-P) compared to nonpregnant rabbits (HEV-NP) was calculated as follows: (HEV RNA copies in HEV-P − HEV RNA copies in HEV-NP)/HEV RNA copies in HEV-NP.

**TABLE 3:** Oligonucleotide primers used in this study

Primer ID^*a*^	Sequence (5′–3′)^*b*^	Purpose
JVHEV-F	GGTGGTTTCTGGGGTGAC	HEV RT-qPCR
JVHEV-R	AGGGGTTGGTTGGATGAA	HEV RT-qPCR
JVHEV-Probe	5′-6-FAM/TGATTCTCAGCCCTTCGC/3′-BHQ-1	HEV RT-qPCR
RPS18-F282	ACTGCCATTAAGGGTGTGGG	qPCR, ribosomal protein S18
RPS18-R425	CTTGTACTGACGCGGGTTCT	qPCR, ribosomal protein S18
IL-1β-F458	GCTCCAGGATGCACAACAGA	qPCR, interleukin 1 beta
IL-1β-R566	ACTCATGGAGAACACCACTTGT	qPCR, interleukin 1 beta
IL2-F7131	GGAAACACAGGAACAACTGGA	qPCR, interleukin 2
IL2-R269	TTTCAATTCTGTGACCTTCTTGG	qPCR, interleukin 2
IL-4-F302	GTCACTCTGCTCTGCCTCCT	qPCR, interleukin 4
IL-4-R302	GCAGAGGTTCCTGTCGAGTCC	qPCR, interleukin 4
IL6-F360	GCCGGCGGTGAATAATGAGA	qPCR, interleukin 6
IL6-R461	TCGTCACTCCTGAACTTGGC	qPCR, interleukin 6
IL-8-113F	GGTACAGAGCTTCGATGCCA	qPCR, interleukin 8
IL-8-281R	CCACTTTTCCTTGGGGTCCA	qPCR, interleukin 8
IL-10-F367	CTGCGACAATGTCACCGATT	qPCR, interleukin 10
IL-10-R487	TGTCAAACTCACTCATGGCT	qPCR, interleukin 10
IL-12A-F206	ATCTTGGGAAAGTCCTGCCG	qPCR, interleukin 12A
IL-12A-R372	TTTGTCTGGCCGCGCA	qPCR, interleukin 12A
IL-12B-F595	GAAGAGAGCCTGCACCTTGA	qPCR, interleukin 12B
IL-12B-R701	GGTGGATCAGGTTTGATGATGT	qPCR, interleukin 12B
IL-13-F295	CACACAACCAGAAGGCTCCAC	qPCR, interleukin 13
IL-13-R407	GTTGCAGCCAGAGACATTGAC	qPCR, interleukin 13
IL-18-F2	TGGCTGCTGAACCAGAAGAG	qPCR, interleukin 18
IL-18-R104	TCTGATTCCAGGTTCTCATCGT	qPCR, interleukin 18
TNF-α-F464	AGCCCACGTAGTAGCAAACC	qPCR, tumor necrosis factor alpha
TNF-α-R563	GTTGTCCGTGAGCTTCATGC	qPCR, tumor necrosis factor alpha
IFN-γ-F	TTCTTCAGCCTCACTCTCTCC	qPCR, interferon gamma
IFN-γ-R	TGTTGTCACTCTCCTCTTTCC	qPCR, interferon gamma
GM-CSF-F198	TCAGAAACCAACTTGCCTGC	qPCR, granulocyte-macrophage colony-stimulating factor^*c*^
GM-CSF-R340	CACAGGAAGTTTCCGGGGT	qPCR, granulocyte-macrophage colony-stimulating factor^*c*^
NF-κB-RelB-F833	TGACCGTGAACGTCTTCCTG	qPCR, NF-κB RelB subunit
NF-κB-RelB-R1025	GGCTTTTTCTTCCGCCGTTT	qPCR, NF-κB RelB subunit
NF-κB-Rel-F1156	CTGTGGCAGGATTGCGTTAAT	qPCR, NF-κB Rel subunit
NFkB-Rel-R1298	TCTGCTTGACTGGAAACCCC	qPCR, NF-κB Rel subunit
PIBF1-F2360	ACGACTAAAGCAGAGTGTCCA	qPCR, progesterone immunomodulatory binding factor 1
PIBF1-R2499	ACAGCGAATTGGCCCTGTTA	qPCR, progesterone immunomodulatory binding factor 1
PRMC1-F410	AAGTTCTATGGACCCGAGGGA	qPCR, progesterone receptor membrane component 1
PRMC1-R491	TATCCAGGCAAAACGTGGCA	qPCR, progesterone receptor membrane component 1
PRMC2-F490	CTCCAGAGGACTGGCAACAT	qPCR, progesterone receptor membrane component 2
PRMC2-R632	TGCCCACATAATCGTATTTTTCTTT	qPCR, progesterone receptor membrane component 2
ESR1-F1049	AGTCTGGTCCTGTGAGGGTT	qPCR, estrogen receptor 1
ESR1-R1228	CGGTCTTTCCGTATACCTTTCA	qPCR, estrogen receptor 1
ESR2-F358	CCATCCGTTGTCAGTCGTCA	qPCR, estrogen receptor 2
ESR2-R463	TTCAGGGCCTCTCTGTTCAC	qPCR, estrogen receptor 2

a F, forward primer; R, reverse primer.

b Primers were designed based on the New Zealand White rabbit nucleotide sequences available on GenBank/NCBI.

c Also known as colony-stimulating factor 2.

### RT-qPCR for quantification of cytokine and pregnancy hormone gene mRNA expression in tissues.

Two-step RT-qPCR assays were developed and optimized to quantify the gene expression levels of a panel of inflammatory cytokines in liver tissues as well as the pregnancy hormone genes in liver and placenta tissues. Briefly, cDNA was synthesized from total RNA extracted from liver and placenta tissues, using high-capacity cDNA reverse transcription kit (Applied Biosystems, Thermo Fisher) and random primers according to the manufacturer’s instructions. The cDNAs were then used to quantify the mRNA expression levels of cytokine and pregnancy hormone genes by using SensiFAST SYBR No-ROX kit (Bioline USA Inc.) and rabbit gene-specific primers (Table 3) designed in this study or elsewhere (86). The qPCR thermal cycling program was as follows: 95°C for 2 min, followed by 40 to 50 cycles of 95°C for 5 s, 60°C for 10 s, and 72°C for 20 s. Melt curve analysis was immediately applied at the end of the amplification cycles in every assay. The rabbit ribosomal protein S18 gene (*RPS18*) was used as a housekeeping gene to calculate the normalized value of threshold cycle (Δ*CT*) for target genes in each rabbit. Cytokine gene mRNA expression levels were normalized in each infected rabbit as follows: ΔΔ*CT* of HEV-P (or -NP) rabbit = Δ*CT* of HEV-P (or -NP) rabbit/mean Δ*CT* of PBS-P (or -NP) group. The same formula was also followed for negative-control rabbits as follows: ΔΔ*CT* of PBS-P (or -NP) rabbit = Δ*CT* of PBS-P (or -NP) rabbit/mean Δ*CT* of PBS-P (or -NP) group. The 2^−ΔΔ^*^CT^* value was then calculated, which reflects the fold change in gene expression in infected rabbit; this value in the control group is expected to be 1.0 but may become higher depending on individual variability within the control group. The fold change in cytokine gene expression after normalization between different groups was calculated as follows: (normalized cytokine gene expression level in group A − normalized cytokine gene expression level in group B)/normalized cytokine gene expression level in group B = fold increase or decrease in group A compared to group B.

### Determination of serum levels of two liver function biomarkers.

The levels of total bilirubin (TBB) and alkaline phosphatase (ALP) in weekly serum samples collected from each rabbit at different time points (0 to 28 dpi) were measured using commercial kits (MyBioSource), rabbit TBB competitive ELISA (catalog no. MBS755725) and rabbit ALP ELISA (catalog no. MBS762814) kits, respectively, according to the manufacturer’s instructions.

### Detection of serum levels of cytokines and pregnancy hormones in rabbits.

A panel of cytokines were assayed in weekly serum samples of each rabbit by using the following rabbit-specific commercial ELISA kits: IL-2, IL-6, and IL-12 (MyBioSource; catalog nos. MBS703067, MBS704834, and MBS728875, respectively) and IFN-γ (Invitrogen rabbit IFN-gamma ELISA kit; Fisher Scientific; catalog no. ER4RB), following the manufacturers’ instructions. The serum levels of pregnancy hormones estrone (E1) and estradiol (E2) in each rabbit were determined using ELISA kits (MyBioSource; catalog nos. MBS7256862 and MBS702805, respectively). The fold change in serum cytokine level between different groups was calculated as follows: (serum cytokine concentration in group A − serum cytokine concentration in group B)/serum cytokine concentration in group B = fold increase or decrease in group A compared to group B.

### Statistical analysis.

All data were analyzed with GraphPad Prism 9.3.1 (GraphPad Software Inc.). The differences between the mean values of two treatment groups were analyzed by two-tailed unpaired Student's *t* test or ordinary two-way ANOVA followed by Tukey’s multiple-comparison test. Some serum samples at some time points had undetectable levels of cytokines, liver enzymes, or hormones and thus were not included in statistical analyses. In this case, data were analyzed by mixed-effects model (restricted maximum likelihood [REML]), followed by Tukey’s test. Statistical correlations were calculated with Pearson’s correlation coefficient test. A *P* value of <0.05 was considered statistically significant.
