# Erratum for Mahsoub et al., “Fetal Loss in Pregnant Rabbits Infected with Genotype 3 Hepatitis E Virus Is Associated with Altered Inflammatory Responses, Enhanced Virus Replication, and Extrahepatic Virus Dissemination with Positive Correlations with Increased Estradiol Level”

**DOI:** 10.1128/mbio.02256-23

**Published:** 2023-10-09

**Authors:** Hassan M. Mahsoub, C. Lynn Heffron, Anna M. Hassebroek, Harini Sooryanarain, Bo Wang, Tanya LeRoith, Guillermo Raimundi Rodriguez, Debin Tian, Xiang-Jin Meng

## ERRATUM

Volume 14, no. 2, e00418-23, 2023, https://doi.org/10.1128/mbio.00418-23. Page 16, line 17 from the bottom: “a significant positive correlation” should read “a significant negative correlation.”

