# Supplementary material for: Fetal Loss in Pregnant Rabbits Infected with Genotype 3 Hepatitis E Virus Is Associated with Altered Inflammatory Responses, Enhanced Virus Replication, and Extrahepatic Virus Dissemination with Positive Correlations with Increased Estradiol Level
Source: mBio. 2023 Mar 20;14(2):e00418-23. doi: 10.1128/mbio.00418-23 (PMC10128027; doi:10.1128/mbio.00418-23)
Supplement: TABLE S1 [file mbio.00418-23-s0003.docx]

**Table S1.** The numbers of kits born alive and the numbers of stillbirths per litter in HEV-3ra-inoculated and mock-inoculated (PBS) pregnant rabbit groups

| Group*^a^* | Rabbit ID# | No. kits born alive/litter | No. stillbirths |
| --- | --- | --- | --- |
| PBS-P | 15 | 7 | 0 |
|  | 17 | 7 | 0 |
|  | 18 | 11 | 0 |
|  | 22 | 8 | 0 |
|  | 35 | 8 | 0 |
|  | 36 | 9 | 0 |
|  | 37 | 9 | 0 |
|  | 38 | 10 | 1 |
| HEV-P | 1 | 8 | 0 |
|  | 5 | 5 | 0 |
|  | 7 | 7 | 0 |
|  | 30 | 5 | 0 |
|  | 31 | 6 | 1 |
|  | 32 | 7 | 0 |
|  | 33 | 9 | 0 |
|  | 34 | 6 | 1 |

*^a^*PBS-P, PBS-inoculated pregnant rabbits; HEV-P, HEV-3ra-infected pregnant rabbits.
