# Supplementary material for: Fetal Loss in Pregnant Rabbits Infected with Genotype 3 Hepatitis E Virus Is Associated with Altered Inflammatory Responses, Enhanced Virus Replication, and Extrahepatic Virus Dissemination with Positive Correlations with Increased Estradiol Level
Source: mBio. 2023 Mar 20;14(2):e00418-23. doi: 10.1128/mbio.00418-23 (PMC10128027; doi:10.1128/mbio.00418-23)
Supplement: TABLE S2 [file mbio.00418-23-s0004.docx]

**Table S2.** Viral RNA load data (log_10_ copies of HEV RNA per gram) in fecal and large intestinal content (LIC) samples collected from HEV-3ra-infected pregnant (HEV-P) and nonpregnant (HEV-NP) rabbits

| Group | Rabbit ID# | Days post-inoculation for fecal samples | | | | | | | | | LIC*^a^* |
| --- | --- | --- | --- | --- | --- | --- | --- | --- | --- | --- | --- |
|  |  | **0** | **3** | **7** | **10** | **14** | **17** | **21** | **24** | **28** |  |
| HEV-P | 1 | 0 | 4.16 | 4.83 | 5.98 | 5.90 | 6.47 | 6.27 | 6.28 | 6.55 | 6.49 |
|  | 5 | 0 | 4.24 | 4.98 | 5.55 | 6.11 | 6.22 | 6.29 | 6.31 | 6.19 | 6.77 |
|  | 7 | 0 | 3.99 | 4.87 | 4.73 | 5.59 | 6.33 | 6.10 | 6.20 | 6.40 | 6.56 |
|  | 30 | 0 | 3.45 | 4.09 | 5.02 | 5.57 | 5.25 | 5.24 | 5.72 | 4.35 | 5.05 |
|  | 31 | 0 | 3.55 | 5.02 | 5.72 | 6.09 | 6.20 | 6.30 | 5.77 | 5.65 | 5.65 |
|  | 32 | 0 | 3.92 | 4.46 | 4.69 | 4.74 | 5.51 | 4.64 | 5.40 | 5.40 | 5.91 |
|  | 33 | 0 | 3.47 | 4.36 | 5.12 | 5.00 | 4.58 | 4.86 | 4.62 | 4.68 | 4.37 |
|  | 34 | 0 | 3.56 | 5.21 | 5.84 | 5.70 | 5.56 | 5.83 | 6.16 | 6.05 | 6.79 |
| HEV-NP | 9 | 0 | 4.18 | 4.79 | 5.39 | 5.58 | 5.54 | 5.66 | 5.81 | 6.30 | 6.37 |
|  | 10 | 0 | 3.61 | 4.70 | 5.08 | 5.52 | 5.77 | 5.65 | 5.48 | 5.54 | 5.90 |
|  | 12 | 0 | 3.42 | 4.39 | 4.65 | 4.83 | 5.40 | 5.56 | 5.10 | 5.44 | 5.47 |
|  | 14 | 0 | 2.56 | 0.00 | 4.27 | 4.50 | 5.18 | 5.48 | 5.28 | 5.23 | 6.00 |
|  | 16 | 0 | 3.15 | 4.38 | 4.70 | 5.23 | 5.34 | 5.80 | 4.81 | 4.69 | 5.02 |
|  | 43 | 0 | 3.75 | 4.86 | 5.46 | 5.73 | 5.51 | 5.93 | 5.52 | 5.84 | 5.44 |
|  | 44 | 0 | 3.32 | 5.53 | 5.91 | 5.56 | 5.66 | 5.29 | 4.76 | 5.40 | 5.77 |
|  | 45 | 0 | 2.72 | 4.57 | 4.56 | 5.26 | 5.42 | 5.25 | 5.08 | 4.96 | 5.79 |

*^a^*Collected at necropsy at 28 dpi.
