# Supplementary material for: Fetal Loss in Pregnant Rabbits Infected with Genotype 3 Hepatitis E Virus Is Associated with Altered Inflammatory Responses, Enhanced Virus Replication, and Extrahepatic Virus Dissemination with Positive Correlations with Increased Estradiol Level
Source: mBio. 2023 Mar 20;14(2):e00418-23. doi: 10.1128/mbio.00418-23 (PMC10128027; doi:10.1128/mbio.00418-23)
Supplement: TABLE S3 [file mbio.00418-23-s0005.docx]

**Table S3.** Viral load data (log_10_ copies of HEV RNA/mL or gram) in serum, bile, and tissue samples collected from HEV-3ra-infected pregnant (HEV-P) and nonpregnant (HEV-NP) rabbits

| Group | Rabbit ID# | Serum sample at indicated dpi*^a^* | | | | | Bile | Liver | Spleen | Ovary | Placenta |
| --- | --- | --- | --- | --- | --- | --- | --- | --- | --- | --- | --- |
|  |  | 0 | 7 | 14 | 21 | 28 |  |  |  |  |  |
| HEV-P | 1 | 0.00 | 3.73 | 3.75 | 3.49 | 3.34 | 10.81 | 9.48 | 7.66 | 7.48 | N/A |
|  | 5 | 0.00 | 0.00 | 0.00 | 3.49 | 0.00 | 9.39 | 9.09 | 7.80 | 5.75 | 3.76 |
|  | 7 | 0.00 | 0.00 | 0.00 | 3.60 | 3.64 | 9.45 | 9.41 | 7.98 | 0.00 | 4.10 |
|  | 30 | 0.00 | 0.00 | 0.00 | 0.00 | 3.02 | 7.43 | 8.06 | 0.00 | 0.00 | 0.00 |
|  | 31 | 0.00 | 3.24 | 3.40 | 3.04 | 3.53 | 9.36 | 8.92 | 6.97 | 6.19 | 0.00 |
|  | 32 | 0.00 | 0.00 | 0.00 | 3.78 | 3.55 | 9.94 | 8.80 | 7.25 | 6.19 | 3.80 |
|  | 33 | 0.00 | 0.00 | 0.00 | 0.00 | 0.00 | 8.72 | 7.36 | 0.00 | 6.96 | 0.00 |
|  | 34 | 0.00 | 0.00 | 0.00 | 3.23 | 3.58 | 9.71 | 8.92 | 0.00 | 7.88 | 0.00 |
| HEV-NP | 9 | 0.00 | 0.00 | 0.00 | 0.00 | 3.22 | 9.54 | 8.91 | 6.83 | 0.00 | N/A |
|  | 10 | 0.00 | 0.00 | 0.00 | 0.00 | 3.15 | 9.37 | 8.70 | 0.00 | 0.00 | N/A |
|  | 12 | 0.00 | 0.00 | 0.00 | 0.00 | 0.00 | 8.61 | 8.23 | 0.00 | 0.00 | N/A |
|  | 14 | 0.00 | 0.00 | 3.29 | 0.00 | 3.25 | 9.49 | 8.16 | 0.00 | 0.00 | N/A |
|  | 16 | 0.00 | 0.00 | 0.00 | 0.00 | 0.00 | 8.17 | 8.31 | 0.00 | 0.00 | N/A |
|  | 43 | 0.00 | 0.00 | 0.00 | 0.00 | 0.00 | 8.84 | 7.82 | 0.00 | 0.00 | N/A |
|  | 44 | 0.00 | 0.00 | 0.00 | 0.00 | 0.00 | 8.22 | 8.01 | 0.00 | 0.00 | N/A |
|  | 45 | 0.00 | 0.00 | 0.00 | 0.00 | 0.00 | 8.53 | 7.38 | 0.00 | 0.00 | N/A |

*^a^*dpi, days post-inoculation.
