# Supplementary material for: Fetal Loss in Pregnant Rabbits Infected with Genotype 3 Hepatitis E Virus Is Associated with Altered Inflammatory Responses, Enhanced Virus Replication, and Extrahepatic Virus Dissemination with Positive Correlations with Increased Estradiol Level
Source: mBio. 2023 Mar 20;14(2):e00418-23. doi: 10.1128/mbio.00418-23 (PMC10128027; doi:10.1128/mbio.00418-23)
Supplement: FIG S2 [file mbio.00418-23-s0002.pdf]

Figure S2

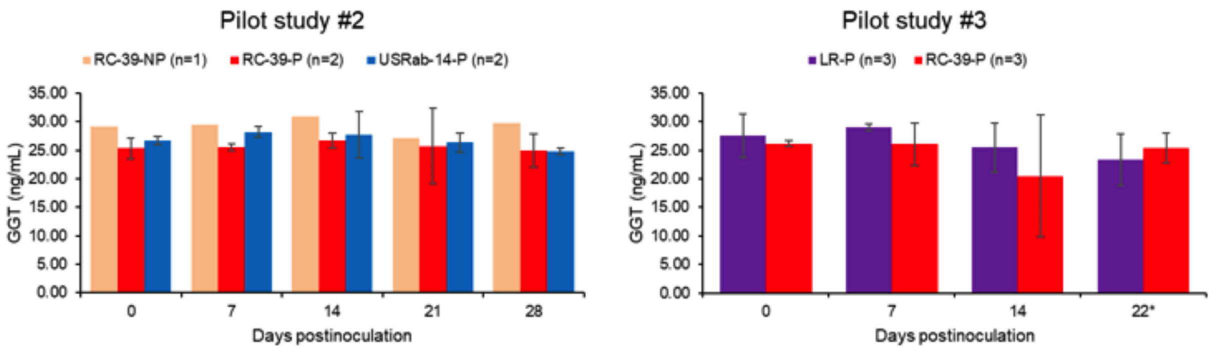

Fig. S2. Pilot animal infection studies in pregnant and non-pregnant rabbits. To optimize the HEV infection study in pregnant rabbits and identify appropriate virus strain, infection timing, and laboratory test parameters, we first conducted three pilot animal studies with a very small number of animals. The serum levels of gamma-glutamyl transferase (GTT) in pregnant and nonpregnant rabbits experimentally infected with three different strains of genotype 3 rabbit HEV (RC-39, USRab-14, and LR) were tested with a Rabbit GGT ELISA kit (MyBioSource; Cat. # MBS1601532). Results are from pilot study #2 (0-28 dpi) and pilot study #3 (0-22 dpi) are showed here. There was no difference in serum level of GTT between infected and control, and between pregnant rabbits and nonpregnant rabbits. Therefore, based on the pilot study result, GTT was not tested in this study. Also, based on collective virology and clinical data from the pilot studies, we chose to use HEV-3ra strain RC-39 for this study. P, pregnant rabbits; NP, nonpregnant rabbits.
