# Supplementary material for: Fetal Loss in Pregnant Rabbits Infected with Genotype 3 Hepatitis E Virus Is Associated with Altered Inflammatory Responses, Enhanced Virus Replication, and Extrahepatic Virus Dissemination with Positive Correlations with Increased Estradiol Level
Source: mBio. 2023 Mar 20;14(2):e00418-23. doi: 10.1128/mbio.00418-23 (PMC10128027; doi:10.1128/mbio.00418-23)
Supplement: TABLE S4 [file mbio.00418-23-s0006.docx]

**Table S4.** Differences in fold change of gene expression levels of selected cytokines/chemokines and pregnancy-related genes in the liver among the four study groups of pregnant and nonpregnant female rabbits experimentally infected with HEV-3ra or mock-infected with PBS

| Gene*^a^* | HEV-P*^b^*  *vs.* | | HEV-NP  *vs.*  PBS-NP | PBS-P  *vs.*  PBS-NP | Determining factor*^c^* |
| --- | --- | --- | --- | --- | --- |
|  | PBS-P | HEV-NP |  |  |  |
| IFN-γ | 8.46 | 0.65 | 4.37 | -0.06 | Infection ↑ |
| TNF-α | 0.83 | 0.03 | 0.79 | 0.01 | Infection ↑ |
| IL-1β | 0.34 | -0.02 | 0.41 | 0.03 | Infection ↑ |
| IL-2 | 0.28 | 0.46 | 0.01 | 0.16 | Pregnancy w/ infection ↑ |
| IL-4 | 0.27 | 0.26 | 0.00 | 0.00 | Pregnancy w/ infection ↑ |
| IL-6 | 0.35 | -0.57(↑NP) | 1.10 | -0.34 | Complicated |
| IL-8 | 0.52 | 1.21 | -0.21 | 0.15 | Pregnancy w/ infection ↑ |
| IL-10 | 0.67 | 0.32 | 0.23 | -0.03 | Pregnancy w/ infection ↑ |
| IL-12A | -0.08 | -0.06 | -0.03 | -0.01 | None |
| IL-12B | -0.29 | -0.17 | -0.12 | 0.03 | Pregnancy w/ infection ↓ |
| IL-13 | -0.01 | 0.03 | -0.12 | -0.09 | None |
| IL-18 | 0.58 | 0.51 | 0.07 | 0.02 | Pregnancy w/ infection ↑ |
| GM-CSF | 0.36 | 0.16 | 0.19 | 0.02 | Infection ↑ |
| NFkB-Rel | -0.11 | -0.14 | 0.03 | 0.00 | None |
| NFkB-RelB | 0.15 | 1.01 | -0.31 | 0.21 | Complicated ↑ / ↓ |
| PIBF1 | -0.08 | 0.23(↓NP) | -0.24 | 0.01 | Nonpregnancy w/ infection ↓ |
| PRMC1 | -0.13 | -0.14 | -0.02 | -0.04 | Pregnancy w/ infection ↓ |
| PRMC2 | -0.10 | 0.24(↓NP) | -0.29 | -0.02 | Nonpregnancy w/ infection ↓ |
| ESR1 | -0.06 | 0.01 | -0.06 | 0.01 | None |
| ESR2 | -0.12 | -0.45(↑NP) | 0.51 | -0.05 | Nonpregnancy w/ infection ↑ |

*^a^*Blue rows are genes with statistically signifcant differences as indicated in main text. Yellow rows are genes with substaial, but not statistcially significant, differences.

*^b^*PBS-NP, mock-infected nonpregnant; PBS-P, mock-infectd pregnant; HEV-NP, HEV-infected nonpregnant; HEV-P, HEV-infected pregnant rabbits.

*^c^*This column shows the factors that have the most effect on gene expresssion; the arrow (up or down) indicates upregulation or downregulation of the specific gene.
