# Supplementary material for: Fetal Loss in Pregnant Rabbits Infected with Genotype 3 Hepatitis E Virus Is Associated with Altered Inflammatory Responses, Enhanced Virus Replication, and Extrahepatic Virus Dissemination with Positive Correlations with Increased Estradiol Level
Source: mBio. 2023 Mar 20;14(2):e00418-23. doi: 10.1128/mbio.00418-23 (PMC10128027; doi:10.1128/mbio.00418-23)
Supplement: FIG S1 [file mbio.00418-23-s0001.pdf]

Figure S1

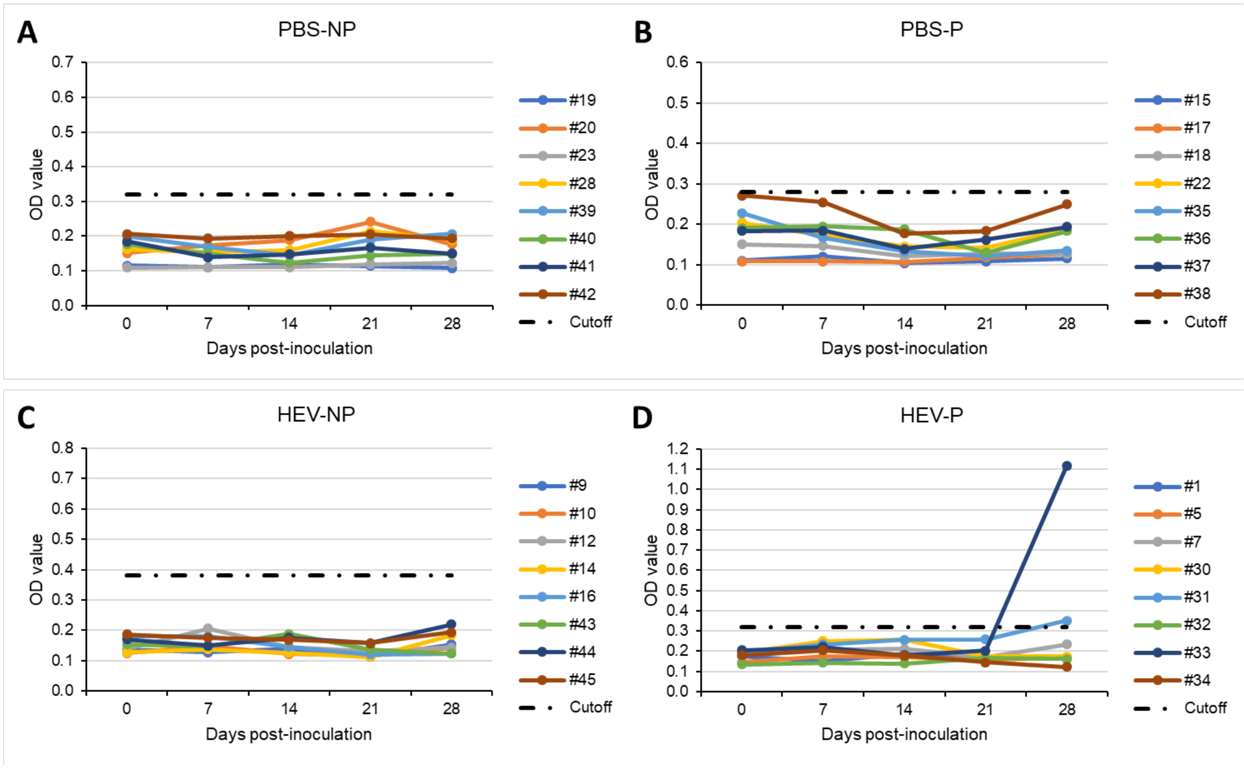

Fig. S1. Anti-HEV IgG antibody responses in HEV-3ra-inoculated or mock-inoculated, pregnant and non-pregnant female rabbits. Individual OD405 values (8 rabbits per group) and cutoff values (dashed line) are plotted. **(A)** PBS-NP, PBS-inoculated non-pregnant rabbits; **(B)** PBS-P PBS-inoculated pregnant rabbits; **(C)** HEV-NP, HEV-inoculated non-pregnant rabbits; **(D)** HEV-P, HEV-inoculated pregnant rabbits. Among the four groups, only two rabbits (ID#31 and #33) in the HEV-infected pregnant rabbit group (**D**) tested seropositive for anti-HEV IgG antibodies at 28 dpi. Rabbit #31 had an OD value that is slightly higher than the cutoff value, while #33 had a very high OD value above the cutoff. The lack of seroconversion in most of the infected rabbits is expected, since the experiment lasted only 28 days. Anti-rabbit HEV IgG antibodies titers were assayed using a HEV ORF2 antigen-based ELISA. Briefly, serum samples were collected weekly from 0 to 28 days post-inoculation (dpi). Serum samples were diluted at 1:200 in blocking buffer. Preimmune and hyperimmune anti-HEV positive rabbit sera were included as negative and positive controls, respectively.
